# Autophagy‐Mediated Suppression of Tumor Growth by Food‐Grade Lipid Nanoparticles in Mice

**DOI:** 10.1002/advs.202504220

**Published:** 2025-07-15

**Authors:** Chenglu Peng, Bing Jiang, Wei Lu, Przemyslaw Zalewski, Jun He, Xiaoyang Li, Yiping Cao, Yiguo Zhao, Cuixia Sun, Katsuyoshi Nishinari, Yapeng Fang

**Affiliations:** ^1^ Department of Food Science and Engineering School of Agriculture and Biology Shanghai Jiao Tong University Shanghai 200240 China; ^2^ School of Health Science and Engineering University of Shanghai for Science and Technology Shanghai 200093 China; ^3^ Department of Pharmacognosy and Biomaterials Poznan University of Medical Sciences Rokietnicka 3 Str. Poznan 60‐806 Poland; ^4^ Department of Food and Human Health Sciences Graduate School of Human Life Science Osaka City University Sumiyoshi Osaka 558‐8585 Japan; ^5^ Glyn O. Phillips Hydrocolloids Research Centre School of Food and Biological Engineering Hubei University of Technology Wuhan 430068 China

**Keywords:** autophagy, food‐grade lipid nanoparticles, lipid droplets, tumor growth

## Abstract

Food‐grade lipid nanoparticles (FLNs) have been widely used as functional carriers of various nutrients and clinical drugs; however, the potential for FLNs to induce substantial biological effects is often overlooked. Here, it is found that FLNs are first delivered to the early endosomes and then preferentially fused with lipid droplets (LDs) after entering the cells through endocytosis. This process leads to a notable LDs accumulation, which in turn triggers autophagy via the AMPK‐mTOR‐ULK1 signaling pathway. The cascade ultimately promotes tumor cell growth and invasion. However, autophagy inhibition while FLNs treatment counteracts these effects and further causes mitochondria damage, increased reactive oxygen species (ROS) levels, and excessive LDs accumulation, eventually leading to cell apoptosis. This indicates a potential anti‐tumor strategy. The animal tests further demonstrate that intratumoral injection of FLNs together with an autophagy inhibitor (3‐MA) effectively suppresses tumor angiogenesis, proliferation, and metastasis without harming normal cells in mice, confirming a promising and safe anti‐tumor strategy of applying FLNs under autophagy inhibition conditions. The findings represent a substantial step forward in comprehending the biological effects of biomedical carriers.

## Graphical abstract

This work reveals a novel extracellular nanoparticle transport and metabolic pathway. Following cellular uptake, Food‐grade lipid nanoparticles (FLNs) selectively fuse with lipid droplets (LDs) rather than lysosomes, driving LDs accumulation. This process activates autophagy through the AMPK‐mTOR‐ULK1 signaling axis, facilitating tumor progression. Autophagy inhibition not only reverses FLNs‐mediated oncogenic effects but also induces mitochondrial dysfunction, elevates reactive oxygen species (ROS), and causes excessive LDs accumulation, culminating in cell apoptosis. These effects collectively suppress tumor angiogenesis, proliferation, and metastatic potential while preserving normal cellular function.

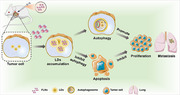

## 1 Introduction

Nanoparticles (NPs) used as functional carriers in biomedical research exhibit a wide range of characteristics, including variations in structure, size, and surface chemistry. This diversity leads to a broad spectrum of interactions at the cellular and organelle levels, many of which are not yet fully understood. The unique interactions of NPs may trigger unexpected secondary biological effects, which can be either beneficial or harmful.^[^ ^1^ ^]^ Understanding how the composition of NPs influences biological systems beyond their intended purpose is crucial for predicting their safety and overall bioeffects.

Food lipid nanoparticles (FLNs) are widely used nanocarriers for delivering numerous bioactive nutrients. They are known for their edibility, ease of preparation, high encapsulation and delivery efficiency, and excellent histocompatibility.^[^ ^2^, ^3^ ^]^ FLNs have also been extensively employed as promising delivery carriers for various nanomedicines in clinical applications,^[^ ^4^, ^5^, ^6^, ^7^ ^]^ particularly in the delivery of macromolecular drugs, including DNA, mRNA, and proteins.^[^ ^8^, ^9^, ^10^, ^11^ ^]^ However, as the non‐payload portion of nanomedicines, FLNs can constitute the majority of the particles. How they interact with biological systems and whether they possess potential negative or positive clinical or therapeutic effects is still little known. The absence of crucial data on the internalization process of FLNs by relevant cells and their subsequent intracellular bioeffects represents a significant knowledge gap that needs to be addressed for further advancements.

Therefore, this study employed monoglyceride‐stabilized soybean oil NPs as a model to investigate the cellular effects of FLNs. Interestingly, a novel metabolism mode of FLNs was observed, in which they were internalized into intestinal cells and fused with intracellular lipid droplets (LDs) rather than undergoing typical lysosomal fusion. This fusion resulted in LDs accumulation and activated autophagy via the AMPK‐mTOR‐ULK1 signaling pathway. Subsequent in vivo experiments validated these in vitro findings and demonstrated that FLNs treatment alone promoted tumor cell growth and metastasis. However, inhibiting autophagy effectively suppressed tumor growth and metastasis in response to FLNs treatment. This dual effect of FLNs on colon tumor growth, mediated through autophagy, highlights their potential in future biomedical applications for cancer therapy. Furthermore, the study revealed a direct interaction between FLNs and the intracellular energy metabolism regulation mechanism of tumor cells via LDs.

## 2 Cellular Uptake of FLNs by Colon Cancer Cells via Clathrin‐ and Caveolae‐Mediated Pathways

To explore the cellular effects of FLNs, spherical 3D lipid NPs were prepared using soybean oil and monoglyceride. The successful fabrication of the FLNs was confirmed using transmission electron microscopy (TEM) and dynamic laser scattering (DLS) (*Appendix*, Figure S1, Supporting Information). They have the same structural properties as the conventional lipid NPs.

Given the ongoing controversy over whether food‐grade NPs can be absorbed in their intact form, we also investigated the internalization of FLNs (*Appendix*, Figures S2 and S3, Supporting Information) in this study. After 2–4 h incubation, Nile red‐labeled FLNs were primarily located on the surface of the cell membrane, with a small fraction (≈20%) internalized within the cells (*Appendix*, Figure S2, Supporting Information). When the incubation time was extended beyond 4 h, a significant number of FLNs were observed entering the cells and dispersing uniformly throughout the cytoplasm (*Appendix*, Figure S3, Supporting Information). Quantitative analysis using flow cytometry revealed that the amount and percentage of uptake increased significantly with higher FLNs concentrations and longer incubation times (*Appendix*, Figure S3, Supporting Information). Further study demonstrated that NaN_3_, chlorpromazine (CPZ), and nystatin (NY) significantly inhibited FLNs uptake (*Appendix*, Figure S4, Supporting Information), indicating that FLNs are primarily absorbed through the energy‐dependent clathrin‐ and caveolae‐mediated endocytosis.

## 3 Intracellular LDs Change upon FLNs Treatment

**Figure 1:**
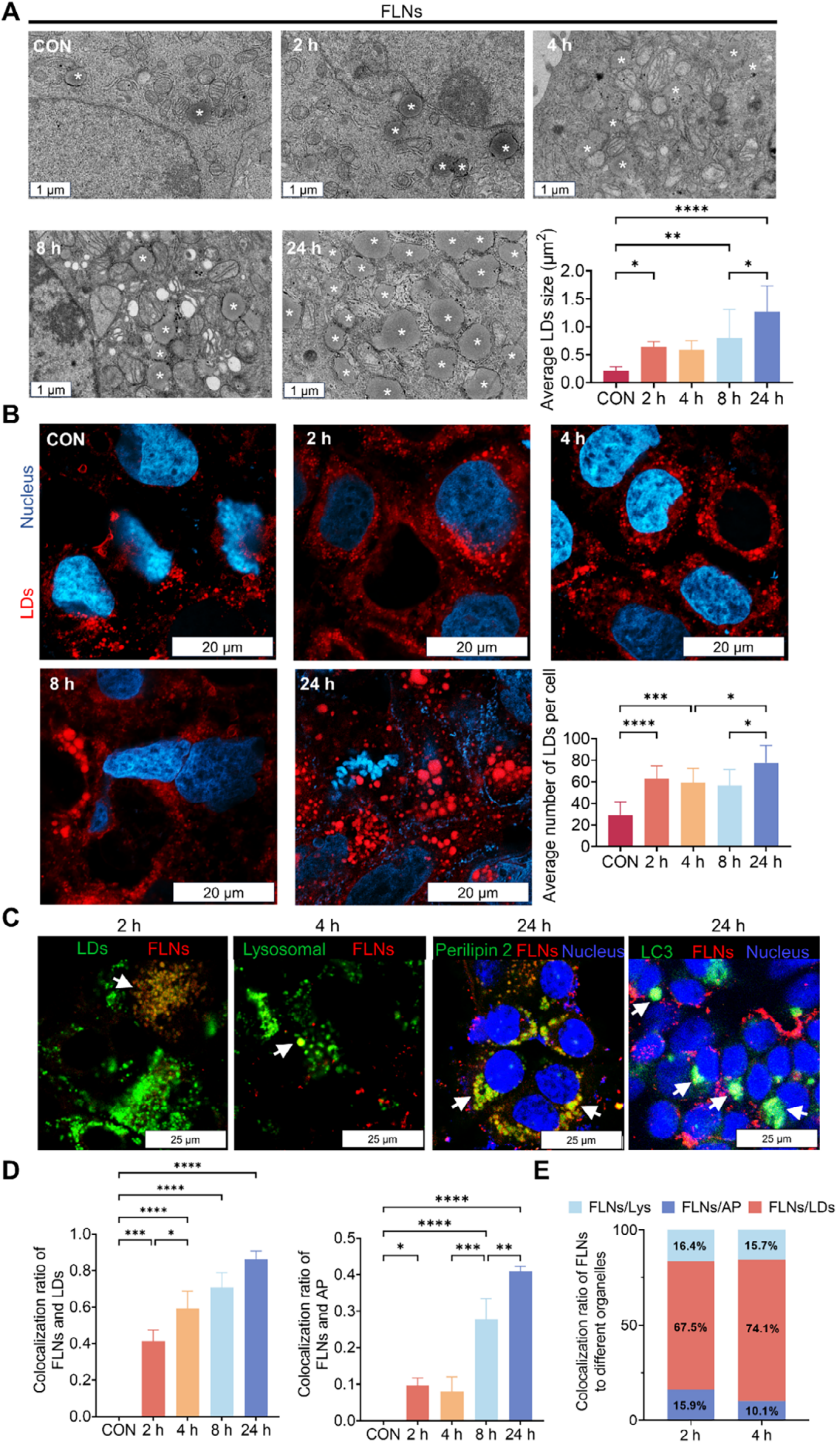
FLNs induced changes in the intracellular LDs structure. A) TEM observation of intracellular LDs. Star (*) is used to signify the LDs. Scale bars: 1 µm. B) CLSM observation of intracellular LDs, DAPI (blue) staining of cell nuclei, and Nile red staining of LDs. Scale bars: 20 µm. C) (from left to right), Colocalization of FLNs (red) with LDs (green), colocalization of FLNs (red) with lysosomes (green), colocalization of FLNs (red) with LDs membrane proteins (green), and colocalization of FLNs (red) with autophagosome (green); white arrows indicate areas of colocalization. Scale bars: 25 µm. D) The colocalization ratio of FLNs with LDs or AP. E) The distribution percentage of FLNs in different organelles (Lys, LDs, and AP). Abbreviations: Lys (Lysosomes), LDs (lipid droplets), AP (Autophagosome). *P < 0.05; **P < 0.01; ***P < 0.001 and ****P < 0.0001. Data are presented as mean ± SD; n=3 independent experiments.

Using TEM and confocal laser scanning microscopy (CLSM), we next showed that FLNs treatment significantly increases the number and average size of intracellular LDs in a time‐dependent manner (**Figure** **1**A,B; *Appendix*, Figure S5, Supporting Information). Since LDs are an organelle that exerts significant influence on the regulation of cellular energy metabolism and immune defense, our investigation aims to elucidate the mechanism underlying LDs accumulation. It is widely recognized that extracellular lipid NPs endocytosed into the cells will first be delivered to lysosomes, where they are degraded. The free fatty acids that are produced are subsequently reassembled into lipid molecules within the endoplasmic reticulum (ER) and are then stored in LDs. Analysis of intracellular transportation tracking revealed that the colocalization of FLNs with LDs became increasingly evident over time, with a noticeable increase in the colocalization ratio (41 ± 6.3%) after 2 h, and reached 86 ± 4.6% after 24 h (Figure 1C,D; Movie S1; *Appendix*; Figure S6, Supporting Information). However, only a small fraction of FLNs exhibited colocalization with lysosomes (10 ± 3.0%) and autophagosomes (9.6 ± 2.0%) at 2 h (Figure 1C,D; Movie S2; *Appendix*, Figures S6 and S7, Supporting Information). The quantitative analysis at 4 h revealed that 74.1% of FLNs were localized within LDs, whereas a smaller proportion was found in lysosomes (15.7%) and autophagosomes (10.1%) (Figure 1E). These results indicate that FLNs exhibit a preference for fusing with LDs rather than lysosomes or autophagosomes, resulting in an augmentation in the size and quantity of intracellular LDs. The findings provide novel insights into the intracellular metabolism of extracellular lipid NPs.

## 4 FLNs Treatment can Trigger Autophagy

**Figure 2:**
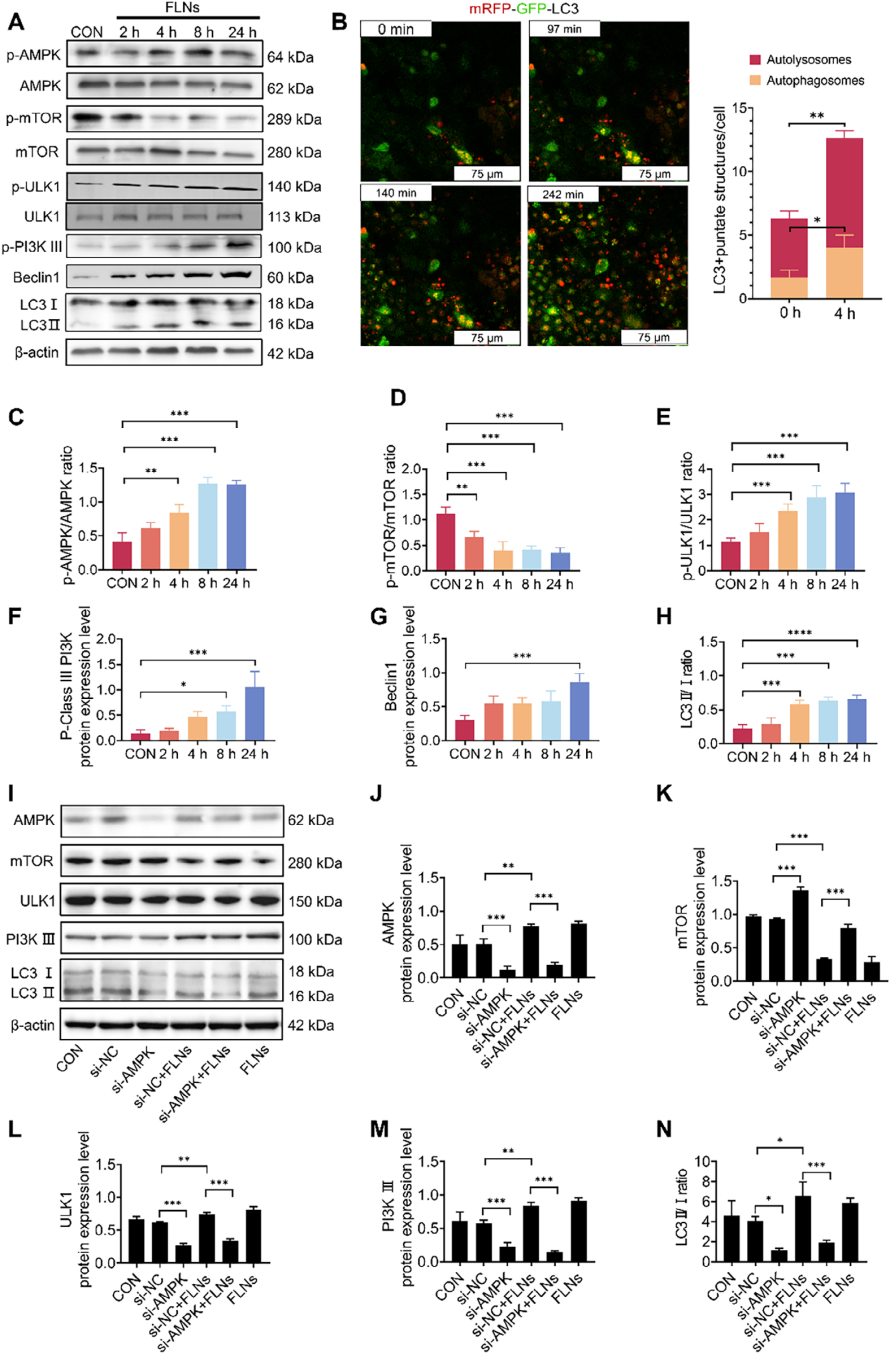
Changes in intracellular AMPK/mTOR/Class III PI3K/Beclin‐1/LC3 protein expression following FLNs treatment. A) Western blot analyses of AMPK and mTOR phosphorylation levels, p‐Class III PI3K and Beclin‐1 protein expression, and LC3II/I ratio. B) Analysis of dynamic autophagy flux using transfected mRFP‐GFP‐LC3 Caco‐2 cell line. Scale bars: 100 µm. C–E) Quantification analysis of phosphorylation levels of AMPK, mTOR, and ULK1, respectively. F–H) Quantification of p‐Class III PI3K and Beclin‐1 protein expression and the LC3II/I ratio. I) Western blot analyses of AMPK, mTOR, ULK1, and Class III PI3K protein expression and LC3II/I ratio. J–N), Quantification of AMPK, mTOR, ULK1, and Class III PI3K protein expression and the LC3II/I ratio. CON: Control group. *P < 0.05; **P < 0.01; ***P < 0.001 and ****P < 0.0001. Data are presented as mean ± SD; n=3 independent experiments.

In addition to the changes in LDs, we also observed a large number of vesicles with a bilayer membrane in FLNs‐treated cells. These vesicles are identified as autophagosomes by subsequent monodansylcadaverine (MDC) staining (*Appendix*, Figure S8, Supporting Information). We then assessed the autophagy level by quantitatively analyzing autophagy indicator proteins, the ratio of microtubule‐associated protein 1 light chain 3 (LC3)‐II/I, before and after FLNs treatment. It was found that the expression of LC3 protein and the LC3‐II/I ratio significantly increased with higher FLNs concentrations, peaking at 300 µg mL^−1^ (*Appendix*, Figure S9, Supporting Information). At the same concentration, the expression of LC3 protein and the LC3‐II/I ratio also significantly increased over time, with the most pronounced effect observed at 4 h (**Figure** **2**A,H; *Appendix*; Figure S10, Supporting Information). Since autophagy is a dynamic process, we constructed a stable transfected cascade monomer RFP‐GFP‐LC3 system (mRFP‐GFP‐LC3) in Caco‐2 cells using lentiviral transfection^[^ ^12^, ^13^ ^]^ to accurately determine the dynamic autophagy flux during the initial 4 h of FLNs treatment. The results showed that the number of punctate autolysosomes and autophagosomes significantly increased over time, specifically, the count went from 1 and 4 at 0 h to 4 and 8 at 4 h per cell (Figure 2B; Movie S3, Supporting Information). These findings provide convincing evidence that FLNs treatment can induce autophagy and the autophagy flux can be enhanced in a dose‐ and time‐dependent manner, with the maximal effect observed at 300 µg mL^−1^. We also prepared FLNs of different diameters (75 ± 5 nm, 150 ± 5 nm, 500 ± 15 nm, and 900 ± 15 nm) (*Appendix*, Figure S11, Supporting Information) and examined the effect of FLNs size on autophagy level. The results indicated that smaller FLNs (d = 75 ± 5 nm and 150 ± 5 nm) significantly increased the autophagic flux compared to larger ones (d = 500 ± 15 nm and 900 ± 15 nm) (*Appendix*, Figure S11, Supporting Information), suggesting that this FLNs‐induced autophagy is size‐dependent. Consequently, all follow‐up investigations utilized FLNs at the optimal concentration of 300 µg mL^−1^ (diameter: 150 ± 5 nm) for consistency. Previous evidence has shown that synthetic metallic or macromolecular NPs can induce autophagy through various mechanisms.^[^ ^14^, ^15^ ^]^ We now demonstrate that food NPs can also induce autophagy, which, to the best of our knowledge, has not been previously reported. Further exploring the potential mechanism of FLNs‐induced autophagy is of great significance.

Interestingly, none of the individual components of FLNs induced an upregulation of autophagy, nor did they significantly promote cell proliferation (*Appendix*, Figure S12, Supporting Information). Furthermore, the effects of various lipid NPs on autophagy levels were evaluated. The findings indicated that sunflower, olive, peanut, and sesame lipid NPs all contributed to increased autophagy levels (*Appendix*, Figure S13, Supporting Information).

It is well known that autophagy is involved in intracellular LDs metabolism.^[^ ^16^ ^]^ We thus explored the relationship between the changes in LDs and variations in autophagy flux. After comparing the changes in LDs and autophagy flux over time, we observed that FLNs‐induced LDs changes (Figure 1A–D) precede the upregulation of autophagy flux (Figure 2A–H), and the accumulation of LDs is significantly enhanced when autophagy is inhibited (*Appendix*, Figure S14, Supporting Information). We thus hypothesize that FLNs induce targeted autophagy, specifically lipophagy, and thereby upregulate the overall autophagy flux. To demonstrate this, TEM observation and colocalization analysis of LDs and autophagosomes were performed. TEM observation demonstrated a higher percentage of LDs‐containing autophagosomes in FLNs‐treated cells compared to the control group (*Appendix*, Figure S14A,D, Supporting Information). Additionally, the colocalization between LDs (red) and autophagosomes (green) was observed, and the level of colocalization significantly increased over time (*Appendix*, Figure S15B,E, Supporting Information). However, when autophagy was inhibited, the colocalization of LDs and autophagosomes (green) was significantly reduced (*Appendix*, Figure S16, Supporting Information).

Based on these observations, we conclude that FLNs first induce an accumulation of LDs in the cytoplasm, which in turn triggers LDs‐targeted autophagy (lipophagy) to catabolize the excess LDs and maintain energy homeostasis. Simultaneously, this process upregulates the level of general autophagy.

Furthermore, we explored the signaling pathway regulation mechanism of FLN‐induced autophagy. The AMP‐activated protein kinase (AMPK) involved signal pathway was analyzed first, as AMPK is well known to be a major regulator of lipid metabolism and plays a critical role in autophagy regulation.^[^ ^17^, ^18^, ^19^ ^]^ AMPK is activated only when phosphorylated, and activated AMPK can upregulate autophagy level through two main pathways: i) inhibiting the mechanistic target of rapamycin (mTOR)^[^ ^20^, ^21^, ^22^, ^23^ ^]^ and thus activating ULK1^[^ ^24^ ^]^ (a key protein in the ULK1 initiation complex of autophagy) through phosphorylation; and/or ii) directly activating ULK1.^[^ ^25^, ^26^, ^27^, ^28^ ^]^ Therefore, the phosphorylation of AMPK and ULK1, and the expression level of mTORC1, were measured. We found that the phosphorylation of AMPK and ULK1 significantly increased, while the expression of mTORC1 was significantly decreased over time following FLNs treatment (Figure 2A–H). Additionally, the expression of two ULK1‐regulated downstream autophagy triggers, Beclin1 and Class III PI3K nucleation complex, was also markedly increased (Figure 2A–H; *Appendix*, Figure S17, Supporting Information).

To further verify the regulatory role of AMPK/mTOR/ULK1 signaling pathway in FLNs‐induced autophagy, we used small interfering RNA (siRNA, siAMPK) to specifically silence *AMPK* gene expression. Silencing of *AMPK* significantly reduced the autophagy levels, as well as the levels of AMPK, ULK1, and Class III PI3K, while increasing mTOR levels in response to FLNs treatment (Figure 2I–N).

In summary, our study demonstrates that FLNs treatment initially induces the accumulation of intracellular LDs, followed by the activation of AMPK. This activation subsequently leads to the inhibition of mTOR, thereby activating the autophagy promoter ULK1 and ultimately initiating the autophagy process.

## 5 FLNs Induce Mitochondrial Damage and Apoptosis

When exploring the intracellular bioeffects of FLNs through TEM observation, it was found that some autophagosomes included mitochondria (*Appendix*, Figure S18, Supporting Information). We therefore further investigated the effect of FLNs on mitochondria, using a staining probe named JC‐1 to detect the mitochondrial membrane potentials (MMP). The results showed that FLNs treatment significantly caused a decrease in MMP after 8 h and this effect was further enhanced after the autophagy of the cells was inhibited (*Appendix*, Figures S19 and S20, Supporting Information), which consequently resulted in a mitochondrial crest rupture and thus the release of Cyt‐c from mitochondria to the cytoplasm (*Appendix*, Figure S21, Supporting Information).

**Figure 3:**
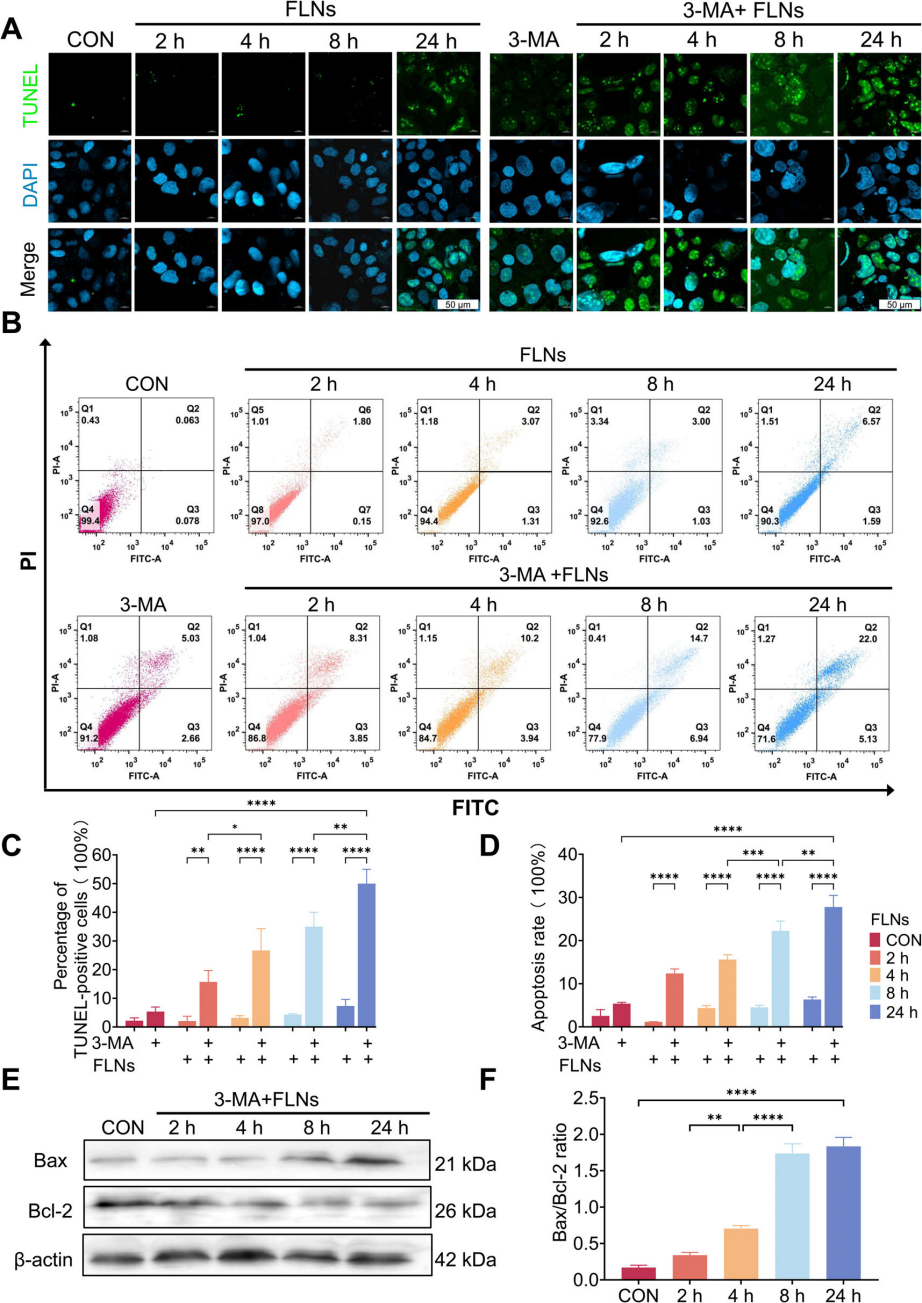
FLNs induced apoptosis of cells after inhibition of autophagy. A) CLSM detection of the effect of FLNs on cell DNA fragmentation before and after inhibition of autophagy (TUNEL staining); DAPI (blue) staining of cell nuclei. Scale bars: 50 µm. B) Flow cytometry detection of cell apoptosis rate in the absence or presence of 3‐MA, followed by FLNs treatment. FITC: fluorescein isothiocyanate; PI: Propidium Iodide. C) Quantitative analysis of the percentage of TUNEL‐positive cells and the rate of apoptosis. D) Quantitative analysis of the apoptosis rate. E) After inhibiting autophagy, FLNs treated cells for different times, Bax and Bcl‐2 protein expression were detected by Western blot. F) Quantitative analysis of Bax / Bcl‐2 ratio. CON: Control group; 3‐MA: 3‐MA treatment for 24 h. *P < 0.05; **P < 0.01; ***P < 0.001 and ****P < 0.0001. Data are presented as mean ± SD; n=3.

To further analyze the cause and the potential subsequent effects of mitochondrial damage, we tested intracellular levels of reactive oxygen species (ROS), IL‐8, and Caspase‐3, as ROS can induce mitochondrial damage and subsequently trigger cell apoptosis.^[^ ^29^ ^]^ We found that intracellular ROS levels significantly increased after 8 h, but IL‐8 and Caspase‐3 levels did not show evident changes (*Appendix*, Figure S22, Supporting Information), indicating that FLNs treatment can significantly increase the intracellular ROS level and thus cause mitochondrial damage but does not induce apoptosis. To further clarify whether FLNs would cause cell DNA damage and induce apoptosis, terminal deoxynucleotidyl transferase dUTP nick‐end labeling (TUNEL) was employed to detect DNA fragmentation, a recognized indicator of apoptosis.^[^ ^30^ ^]^ We found that FLNs treatment didn't significantly increase the number of TUNEL‐positive cells, suggesting that FLNs do not cause significant DNA damage or cell apoptosis (**Figure** **3**).

In contrast, when autophagy was pre‐inhibited with an autophagy inhibitor (3‐MA), the administration of FLNs led to a subsequent increase in ROS, IL‐8, and Caspase‐3 levels (*Appendix*, Figure S22, Supporting Information). Furthermore, FLNs‐treated cells exhibited a significant increase in DNA fragmentation (Figure 3A,C) and apoptosis rates (Figure 3B,D). Based on these findings, we concluded that FLNs treatment initially causes cellular LDs accumulation, which subsequently triggers autophagy, thereby promoting lipid metabolism. If the autophagy is blocked, FLNs treatment can exacerbate oxidative stress, resulting in mitochondrial membrane and DNA damage, leading to intensive cell apoptosis. Similar conclusions have been reached in previous studies, demonstrating that autophagy plays a critical role in alleviating cellular damage by degrading damaged proteins and organelles, such as regulating oxidative stress through the recycling of damaged ROS‐producing mitochondria.^[^ ^31^ ^]^

The mechanism of FLNs‐induced apoptosis following autophagy inhibition was also verified. The findings of this study indicate that after autophagy inhibition, treatment with FLNs significantly increases the level of the pro‐apoptosis‐related protein Bax, while notably reducing the expression level of the anti‐apoptosis‐related protein Bcl‐2 (Figure 3E,F). This suggests that, when autophagy is inhibited, FLNs can induce DNA damage and apoptosis by upregulating Bax expression and downregulating Bcl‐2 expression. This finding can also be considered as a potential cause of the decreased mitochondrial membrane potential and subsequent Cyt‐c release induced by the combination of autophagy inhibitors and FLNs. Because Bax has been recognized for its ability to enhance mitochondrial membrane permeability, leading to the release of Cyt‐c into the cytoplasm.^[^ ^32^ ^]^

## 6 FLNs Promote the Invasion and Migration of Colon Cancer Cells

**Figure 4:**
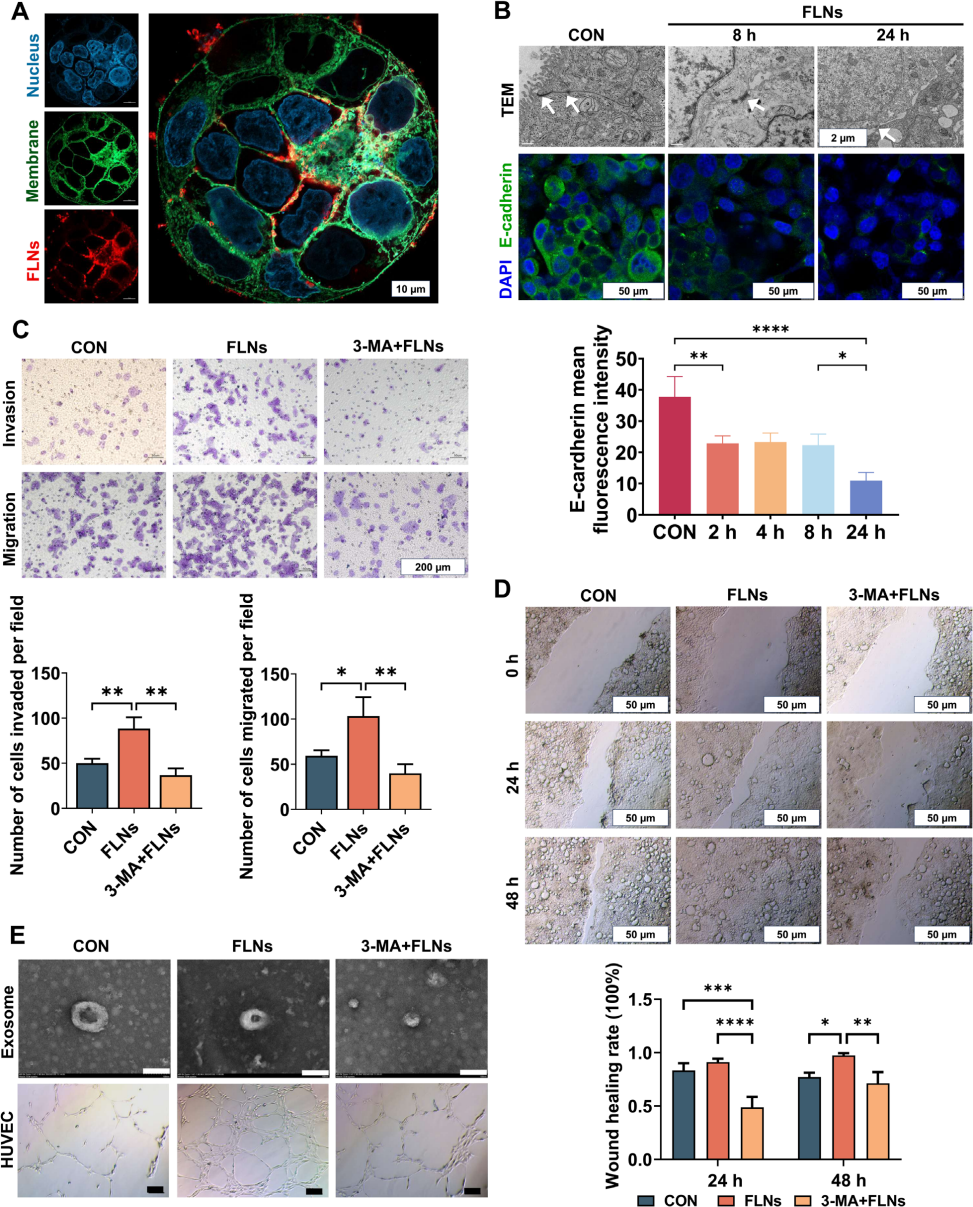
Effect of FLNs on cell invasion, migration, and healing. A) CLSM of FLNs localization, Nile red (red) labeled FLNs, DAPI (blue) stained cell nuclei, Dio (green) stained cell membrane. Scale bars: 10 µm. B) TEM observed intercellular connection (above), scale bars: 2 µm; CLSM observation of E‐cadherin protein expression (below), DAPI (blue) staining of the nucleus, E‐Cadherin positive (green), scale bars: 50 µm. C) Transwell detection of cell invasion and migration. Scale bars: 200 µm. D) The effect of FLNs on wound healing of cells. Scale bars: 50 µm. E) Effects of exosomes on angiogenesis in different treatment groups. White scale bars: 100 nm; Black scale bars: 50 µm. *P < 0.05; **P < 0.01; ***P < 0.001 and ****P < 0.0001. Data are presented as mean ± SD; n=3.

In addition to the bioeffects described above, we observed another intriguing phenomenon: FLNs were found to be enriched at the cell‐cell junctions (**Figure** **4**A). A closer structural characterization by TEM revealed that the intercellular junctions weakened after FLNs treatment, suggesting a regulatory effect of FLNs on cell junctions. This was supported by an Immunofluorescence assay that showed a significant decrease in the expression of E‐cadherin, a core component associated with epithelial adhesion junctions (Figure 4B).

Since intercellular junctions are closely related to cell mobility, we next assessed the effect of FLNs treatment on the invasion and migration of colon cancer cells by Transwell assay. After 48 h incubation, a significant increase in the number of invading cells was found in the FLNs‐treated group, yet such a change was not observed in 3‐MA‐pretreated cells (Figure 4C). The results for cell migration were consistent with those for cell invasion, showing a similar pattern (Figure 4C). The cell scratch test results indicated that FLNs significantly enhanced cell wound healing, while 3‐MA combined with FLNs significantly inhibited wound healing (Figure 4D). Taken together, FLNs treatment can significantly loosen intercellular adhesion by reducing the expression of E‐cadherin, leading to increased cell migration and invasion, suggesting that FLNs treatment potentially can promote cancer cell metastasis. Notably, this effect can be regulated by autophagy. The possible mechanisms include: i) Inhibition of autophagy reduces the degradation rate of accumulated LDs, thereby limiting the energy supply required for cell invasion and migration^[^ ^33^ ^]^; ii) The increased autophagy flux induced by FLNs degrades more E‐cadherin proteins, leading to reduced cell surface E‐cadherin level and thus weakening the tumor metastasis suppressor effect of E‐cadherin.^[^ ^34^ ^]^

Moreover, we also evaluated another cancer‐related indicator: angiogenic ability. Exosomes from cells before and after FLNs treatment were collected and incubated with human umbilical vein endothelial cells (HUVECs). Quantitative analysis demonstrated that exosomes derived from FLNs‐treated cells significantly enhanced angiogenic parameters by: (1) increasing total tube length by 1.3‐fold, (2) elevating branch points by 1.4‐fold, and (3) expanding mesh formation area by 1.5‐fold compared to control groups (Figure 4E; Appendix; Figure S23, Supporting Information). The combination of FLNs with 3‐MA inhibited angiogenesis (Figure 4E; *Appendix*; Figure S23, Supporting Information), suggesting that FLNs treatment enhances the pro‐angiogenic ability of exosomes. Subsequently, we investigated the impact of varying concentrations of FLNs on the viability of diverse cell types (NCM460, RAW264.7, Caco‐2, HeLa, HT29) (*Appendix*, Figure S24, Supporting Information). Additionally, we determined the optimal FLNs concentration for further experiments. Specifically, 300 mg L^−1^ was chosen for Caco‐2, HeLa, and HT29, while 600 mg L^−1^ was selected for NCM460 and RAW264.7 to assess the impact of FLNs combined with 3‐MA on cell viability. The findings indicated that FLNs in conjunction with 3‐MA effectively suppressed the activity of tumor cells (Caco‐2, HeLa, HT29) without significantly affecting the viability of normal cells (NCM460, RAW264.7) (*Appendix*, Figures S25 and S26, Supporting Information).

The results obtained in this section highlight that food‐derived NPs may have a positive effect on tumor metastasis and growth, providing valuable insights for the scientific dietary management of cancer patients.

## 7 In Vivo Validation of the Effects of FLNs on Colon Tumor

**Figure 5:**
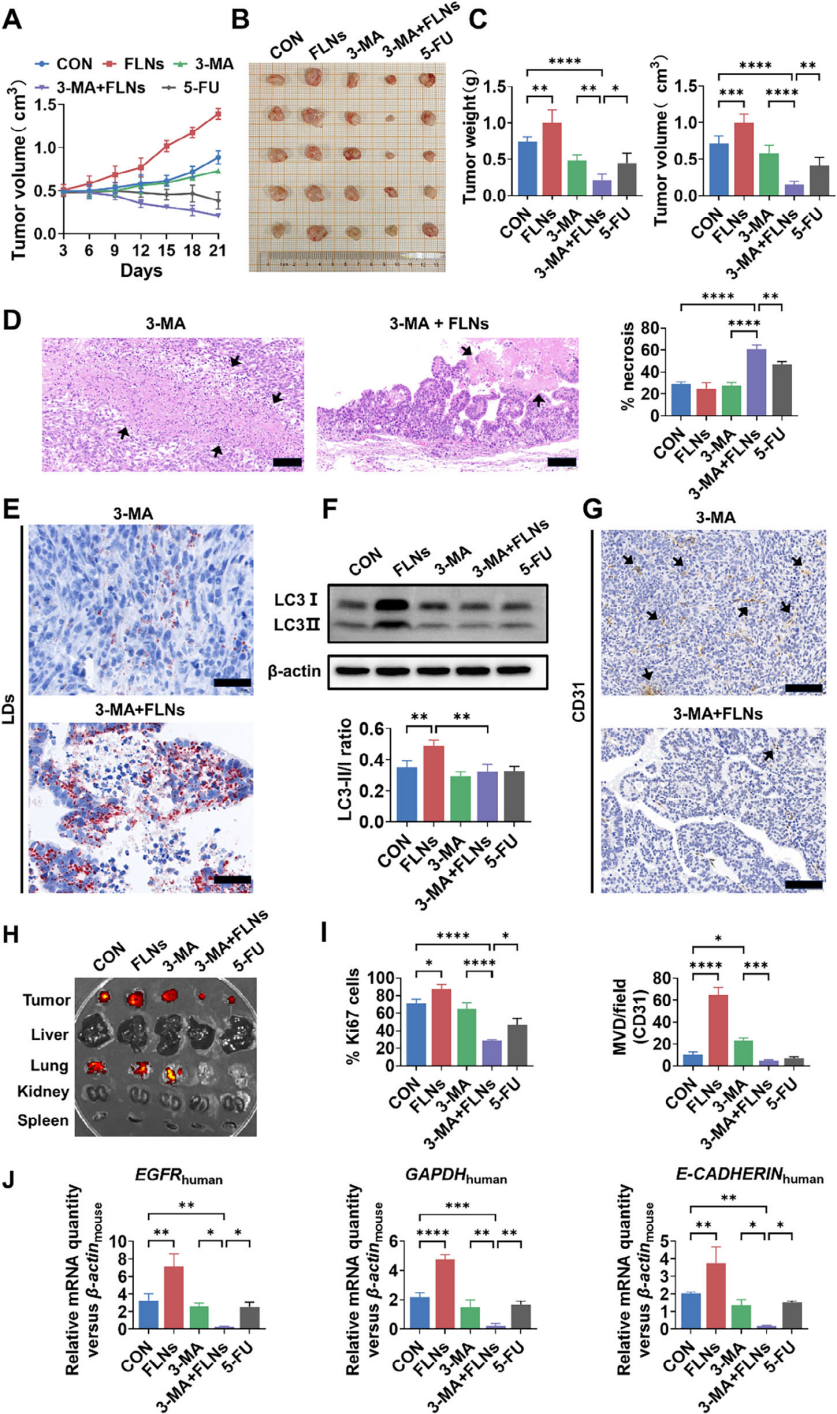
Effect of FLNs on tumor proliferation, metastasis, and angiogenesis in vivo. Intratumoral injections were administered every other day with the following treatments: PBS (100 µL), FLNs (100 µL; 1 mg mL^−1^), 3‐MA (100 µL; 2 mg mL^−1^), FLNs (100 µL; 1 mg mL^−1^) + 3‐MA (100 µL; 2 mg mL^−1^). A) Statistical analysis of the tumor volumes, which were measured every three days and plotted individually. B) Subcutaneous Caco‐2 xenograft tumors formed from different groups were dissected, and a representative image of these xenograft tumors is presented. C) Volume and weight of Caco‐2 xenograft tumors. D) The pathological section of tumor tissue was observed and the necrotic area of tumor was quantitatively analyzed. The black arrows indicate the necrotic area of the tumor; Scale bars: 100 µm. E) LDs in tumor tissue were observed by oil red O staining; red indicates LDs; Scale bars: 50 µm. F) Effects of FLNs on autophagy levels in tumor tissues. G) Sections of tumors stained for CD31. the black arrows indicate positive expression; Scale bars: 50 µm. H) Near‐infrared fluorescence imaging was used to assess tumor metastasis. I) Quantitative analysis of Ki67 positive rate, and microvascular density. J) Quantitative analysis shows changes of *EGFR*_human_, *GAPDH*_human_ and *E‐CADHERIN*_human_ mRNA expression in mice blood samples. Mouse β‐actin is used as a housekeeping gene. *P < 0.05; **P < 0.01; ***P < 0.001 and ****P < 0.0001. Data are presented as mean ± SD; n=5.

Xenograft mouse axillary subcutaneous tumor models were employed to confirm the effect of combined therapy using FLNs treatment and autophagy inhibition on tumor progression in vivo. As expected, following only three weeks of intratumoral injection with FLNs, there was a significant increase in tumor weight and volume. However, the combination treatment of FLNs with 3‐MA significantly reduced both the tumor weight and volume. The administration of 3‐MA alone does not significantly affect tumor size, and in vitro testing has confirmed that FLNs do not modify the cellular uptake of 3‐MA (**Figure** **5**A–C; *Appendix*, Figure S27, Supporting Information). Hence, the combined therapeutic effect observed with FLNs and 3‐MA is not attributed to alterations in the absorption of 3‐MA. Moreover, this combination therapy enhanced oxidative stress and inflammatory responses (MDA, ROS, and TNF‐α) in the tumor tissue (Figure 5A–D; *Appendix*, Figure S28, Supporting Information). Additionally, 5‐FU significantly elevated oxidative stress and inflammation indicators (such as MDA and ROS) in the serum of mice. In contrast, the combination treatment of 3‐MA and FLNs did not significantly increase these oxidative stress indicators in the mice's serum (*Appendix*, Figure S29, Supporting Information). This suggests that the combined treatment of 3‐MA and FLNs has the potential for fewer side effects on the health of mice compared to 5‐FU.

Subsequent investigations revealed that FLNs prompted the intracellular accumulation of LDs (Figure 5E; *Appendix*, Figure S30, Supporting Information) and triggered autophagy in tumor cells in mice (Figure 5F). As described above, the in vitro tests showed that upon autophagy inhibition, FLNs cause excessive accumulation of LDs, ultimately resulting in tumor necrosis in vivo (Figure 5D). Microvessel density and tumor‐promoting factor (Ki67 protein) analysis showed that FLNs treatment significantly enhanced angiogenesis in the tumor tissue and the proliferation of tumor cells. However, FLNs plus 3‐MA treatments effectively inhibited these effects (Figure 5G,I; *Appendix*, Figures S31 and S32, Supporting Information). These results suggested that FLNs can promote tumor growth by accelerating tumor cell proliferation and tumor tissue angiogenesis, which is regulated by autophagy.

Next, the effect of FLNs on tumor cell metastasis in vivo was investigated since our in vitro test showed an enhanced cell invasion and migration capability by FLNs treatment. The tumor cells were fluorescently labeled and tumor metastasis in mouse organs (liver, lung, kidney, and spleen) was evaluated by near‐infrared (NIR) imaging analysis. The results showed that FLNs significantly promoted metastasis to the lungs, and this was completely restrained by the combined use of 3‐MA (Figure 5H). Since Caco‐2 is a human‐derived cell line, the human‐originated gene expression (*EGFR*_human_, *GAPDH*_human,_ and *E‐CADHERIN*_human_) in the mice blood was measured to further determine the metastasis. The results showed that the expression of these human‐originated genes was significantly increased in the mice's blood after FLNs treatment, but this expression was inhibited in the case of autophagy inhibition (Figure 5J).

HE staining was further utilized to verify metastasis through the bloodstream. Numerous tumor cells were detected in the vessels and tissues of the lungs in the FLNs‐treated group, whereas only a scant few cells were present in the lungs of the 3‐MA and 5‐FU‐treated groups. Practically no tumor cells were observed in the group treated with a combination of FLNs and 3‐MA (*Appendix*, Figure S33, Supporting Information). These findings ultimately suggest that FLNs can facilitate tumor cell metastasis to the lungs via blood circulation, a process that is modulated by autophagy.

Moreover, the pathological observations indicated that the combined treatment of 3‐MA and FLNs did not cause significant damage to the lungs, liver, intestines, and spleen. Specifically, the lung tissue structure remained clear without obvious inflammation or fibrotic changes; the liver cells were neatly arranged without necrosis or steatosis; the intestinal mucosa remained intact with normal villus structure and no inflammatory infiltration; and the spleen also showed no significant pathological changes. In contrast, pathological section observation showed that 5‐FU treatment resulted in thickening of alveolar walls in the lungs, various degrees of necrosis in liver cells accompanied by inflammatory cell accumulation, damage to intestinal villus structure, and structural disorder of white and red pulp in the spleen (*Appendix*, Figures S33–S36, Supporting Information). All of these results suggest that the combined use of FLNs with an autophagy inhibitor is expected to provide a novel anti‐tumor strategy with low side effects.

## 8 Discussion and Conclusion

The study on the cellular uptake and intracellular bioeffects of NPs in food is crucial to the clarification of their safety and potential functionalities in vivo. In this study, we found that FLNs can enter into the cells through different endocytosis pathways and caused three main bioeffects: i) fuse with intracellular LDs and leads to an increase in their number and size; ii) induce autophagy; and iii) loosen the intestinal cell adhesion junctions and promote cell migration and invasion. Similar results were also observed in the in vivo test. Among these effects, the changes in LDs induced by FLNs acted as a trigger for autophagy, which played a central role in regulating LDs metabolism and the growth behavior of tumor cells. We found that FLNs promoted tumor proliferation and metastasis. When autophagy was inhibited, FLNs significantly suppressed tumor proliferation and induced tumor cell apoptosis. This tumor suppression effect was comparable to 5‐FU, with fewer toxic side effects than 5‐FU.

LDs are lipid‐rich organelles that regulate the intracellular lipid storage and metabolism. Recent studies have shown that LDs can form the first defensive barrier and act as a molecular switch in innate immunity, responding to danger signals by reprogramming cell metabolism and triggering protein‐mediated antimicrobial mechanisms.^[^ ^4^, ^5^, ^6^, ^7^, ^35^ ^]^ Additionally, LDs accumulation in non‐adipocytic tissues has emerged as a new hallmark of cancer.^[^ ^36^ ^]^ This accumulation, coupled with the catabolism of LDs, is intricately linked to energetic metabolism and cell signaling, thereby significantly influencing cancer cell proliferation, resistance to cell death, and overall aggressiveness.^[^ ^37^ ^]^ Furthermore, studies have also confirmed that the accumulation of LDs enhances lymph angiogenesis and epithelial‐mesenchymal transition of tumor cells, leading to lymph node metastasis in cervical cancer.^[^ ^38^ ^]^ However, the direct fusion of extracellular particles with intracellular LDs has not been previously reported. Our finding that FLNs fusion with LDs provides a potential food‐based strategy to regulate LDs‐mediated cellular immune defense and tumor progression, which may have potential value for clinical nutrition and human health.

Autophagy is a fundamental degradation mechanism that affects lipid homeostasis in several different ways.^[^ ^39^ ^]^ Our results demonstrate that FLNs can induce lipophagy and upregulate overall autophagy flux through AMPK‐mTOR/ULK1‐PI3K III signaling pathway. When the concentration of FLNs increased to a certain level, autophagy flux reached an upper limit, and further increases in FLNs concentration led to cell apoptosis. However, this effect was not observed in FLNs‐treated non‐tumor cells (*Appendix*, Figure S37, Supporting Information), suggesting that FLNs can specifically cause excessive accumulation of LDs and induce apoptosis in tumor cells but not in normal cells. This finding could potentially be developed into a tumor treatment strategy through local injection of FLNs, and our subsequent in vivo tests confirmed the effectiveness of this approach (Figure 5).

FLNs treatment reduces E‐cadherin expression, disrupting intercellular adherens junctions to promote in vitro cell migration/invasion and in vivo colon tumor metastasis. Crucially, autophagy activation mediates E‐cadherin degradation, and autophagy inhibition fully reverses FLNs‐induced migratory, invasive, and angiogenic effects.^[^ ^40^, ^41^ ^]^ Additionally, extracellular vesicles from FLNs‐treated cells enhance HUVEC angiogenesis, facilitating tumor vascularization.^[^ ^42^, ^43^, ^44^ ^]^ These findings suggest that prolonged exposure to FLNs may exacerbate tumor progression by promoting metastasis and angiogenesis. FLNs may also increase intestinal barrier permeability,^[^ ^45^ ^]^ potentially altering nutrient absorption.^[^ ^46^, ^47^ ^]^

In conclusion, our findings offer valuable insights into food‐body interactions, suggesting guidelines for dietary lipid ingestion in healthy and cancer populations. They also highlight the need to reassess the safety of NPs in food. Furthermore, combining FLNs with autophagy inhibitors may offer a promising anti‐tumor strategy with low side effects. Given the tumor‐targeting capability of FLNs,^[^ ^48^ ^]^ their high intestinal absorption efficiency (up to 82%)^[^ ^49^ ^]^ and the successful internalization by colon tumor cells (Figure 4A,B; *Appendix*, Figure S2, Supporting Information), we hypothesize that co‐administering FLNs and autophagy inhibitors orally may exhibit potent antitumor efficacy. Future studies will systematically evaluate the therapeutic efficacy of this combinatorial regimen in orthotopic colon cancer models.

## 9 Experimental Section

Human colon cancer cells HT‐29 and Caco‐2 were purchased from Procell Life Science & Technology Co. Ltd (Wuhan, China). Viable Caco‐2 cells (5 × 10^6^ /200 µL) were subcutaneously injected into the left axilla of 8‐week‐old male M‐NSG mice (6 mice per group). All in vivo experimental protocols were approved by the Animal Protection Committee of Shanghai Jiao Tong University (Ethical Number: A2023175‐001). More details are available in Supporting Information *Appendix*, which includes additional methods, further notes to *Appendix*, Figures S1–S37 (Supporting Information).

### Statistics

The fluorescence colocalization was analyzed via the ImageJ plugin Scatter J (NIH, Bethesda, MD, USA). Significant differences among groups were measured by GraphPad Prism 9. Comparisons between multiple groups were made using one‐way analysis of variance (ANOVA) with Dunnett's multiple comparisons test. For comparisons between two treatments across multiple groups, a two‐way ANOVA with Sidak's multiple comparisons test was used. The data were expressed as the mean ± standard deviation. Significant differences were determined at the 0.05 level (*P* < 0.05).

## Conflict of Interest

The authors declare no conflict of interest.

## Author Contributions

Y.F. and W.L. conceived and designed the study. C. P. performed the experiments and collected the data. B.J. assisted with a portion of the experiments. W.L. and C.P. analyzed the data and wrote the manuscript. P.Z., J.H., K.N., X.L., Y.C., Y.Z., C.S., and Y.F. reviewed and revised the manuscript. W.L. and Y.F. supervised and guided the study.

## Supporting information



Supporting Information

Supplemental Movie 1

Supplemental Movie 2

Supplemental Movie 3

## Data Availability

All data that support the findings of this study are available from the corresponding authors depending on reasonable request.
