# Supplementary material for: Autophagy‐Mediated Suppression of Tumor Growth by Food‐Grade Lipid Nanoparticles in Mice
Source: Adv Sci (Weinh). 2025 Jul 15;12(35):e04220. doi: 10.1002/advs.202504220 (PMC12462971; doi:10.1002/advs.202504220)
Supplement: Supplementary file 1 — Supporting Information [file ADVS-12-e04220-s002.docx]

**Supplementary Information for**

**Autophagy-Mediated Suppression of Tumor Growth by Food-Grade Lipid Nanoparticles in Mice**

Chenglu Peng^1^, Bing Jiang^1^, Wei Lu^1^*, Przemyslaw Zalewski^3^, Jun He^1^, Xiaoyang Li^1^, Yiping Cao^1^, Yiguo Zhao^1^, Cuixia Sun^1^, Katsuyoshi Nishinari^4,5^, Yapeng Fang^1,2^*

^1^Department of Food Science and Engineering, School of Agriculture and Biology, Shanghai Jiao Tong University, 200240, Shanghai, China

Yapeng Fang

^2^School of Health Science and Engineering, University of Shanghai for Science and Technology, Shanghai 200093, China

^3^Department of Pharmacognosy and Biomaterials, Poznan University of Medical Sciences, Rokietnicka 3 Str., 60-806 Poznan, Poland

^4^Department of Food and Human Health Sciences, Graduate School of Human

Life Science, Osaka City University, Sumiyoshi, Osaka, 558-8585, Japan

^5^Glyn O. Phillips Hydrocolloids Research Centre, School of Food and Biological Engineering, Hubei University of Technology, Wuhan, 430068, China

* Corresponding authors

E-mail: [wei.lu@sjtu.edu.cn](mailto:wei.lu@sjtu.edu.cn) & [ypfang@sjtu.edu.cn](mailto:ypfang@sjtu.edu.cn)

Tel: +86 21 34208548

**This PDF file includes:**

SI Appendix, Figure S1–S37

Additional material & methods

Caption for Movie S1-S3

**Figure S1-S36**


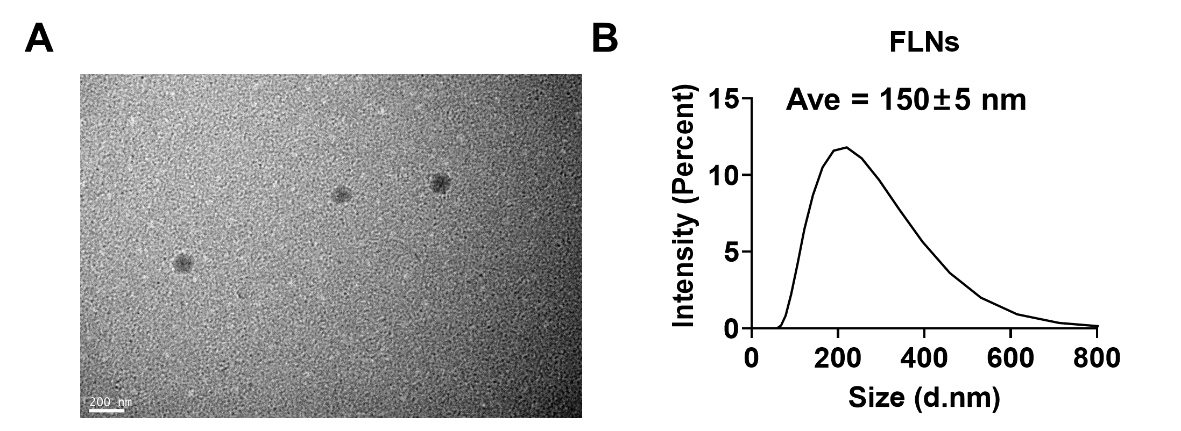


**Figure S1.** The characterization of FLNs. **A)**, the morphology of FLNs was observed by TEM. **B****)**, The average particle size (diameter) and size distribution of FLNs were detected by DLS. Scale bars: 200 nm. Data are presented as mean ± SD (n=3).


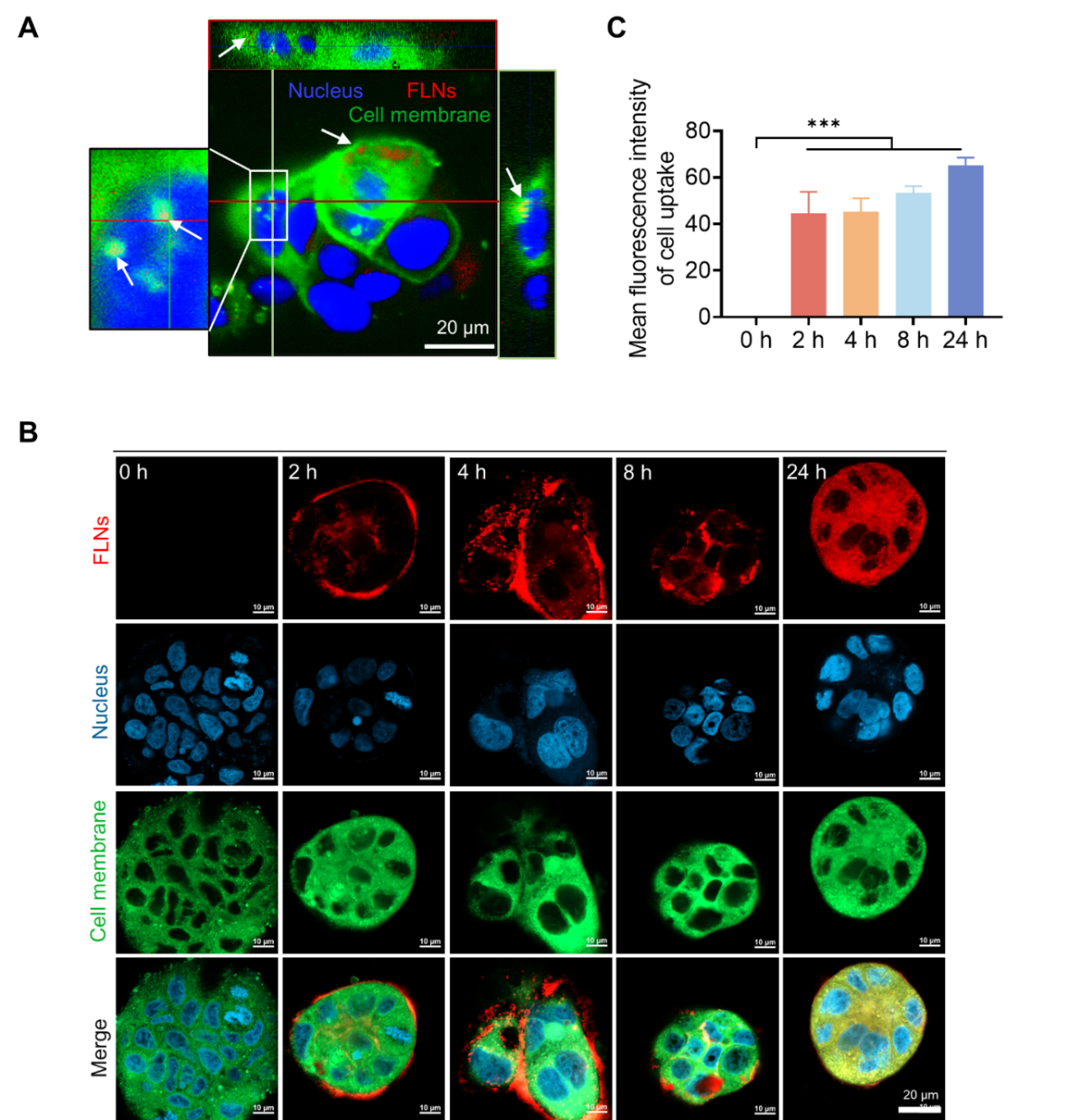


**Figure S2.** CLSM detection of cellular uptake of FLNs. **A)**, The observation of FLNs cellular uptake and their distribution in cells was conducted using the Z-stack mode of CLSM. Red fluorescence indicates Nile red-labelled FLNs. Blue fluorescence indicates DAPI-stained nuclei. Green fluorescence indicates Dio-stained cell membrane, and white arrows indicate FLNs. **B)**, CLSM observation of the cellular absorption of FLNs at different times. **C)**, Quantitative analysis of absorption dose of FLNs based on intracellular Nile red fluorescence intensity. **P* < 0.05; ***P* < 0.01; ****P* < 0.001 and *****P* < 0.0001. Scale bars: 20 µm. Data are presented as mean ± SD (n=3).


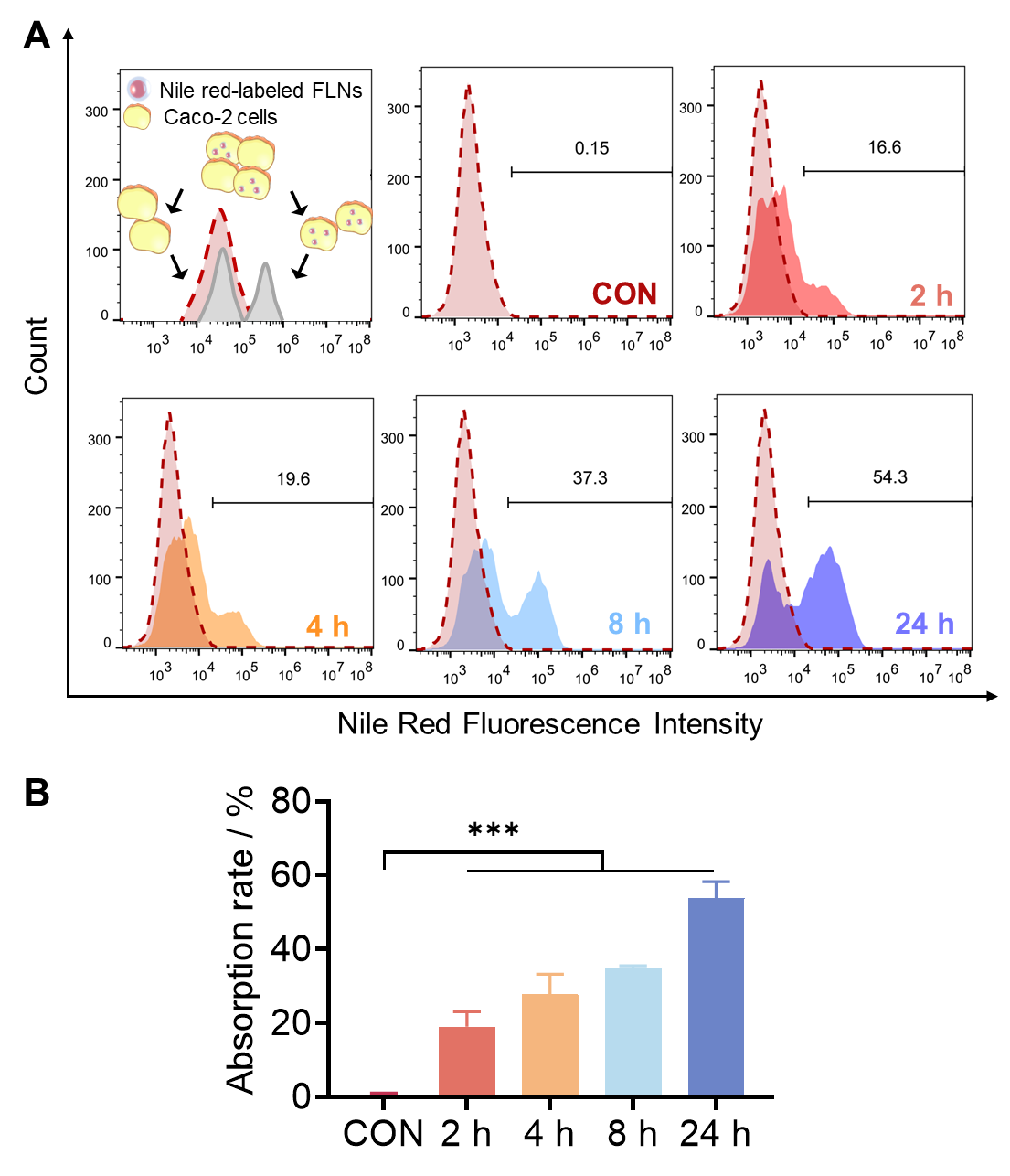


**Figure S3.** Flow cytometry detection of cellular uptake of FLNs. **A)**, Flow cytometric diagrams of cellular uptake of FLNs at different times. **B)**, Quantitative analysis of FLNs uptake by cells at different times was detected by flow cytometry. **P* < 0.05; ***P* < 0.01; ****P* < 0.001 and *****P* < 0.0001. Data are presented as mean ± SD (n=3).


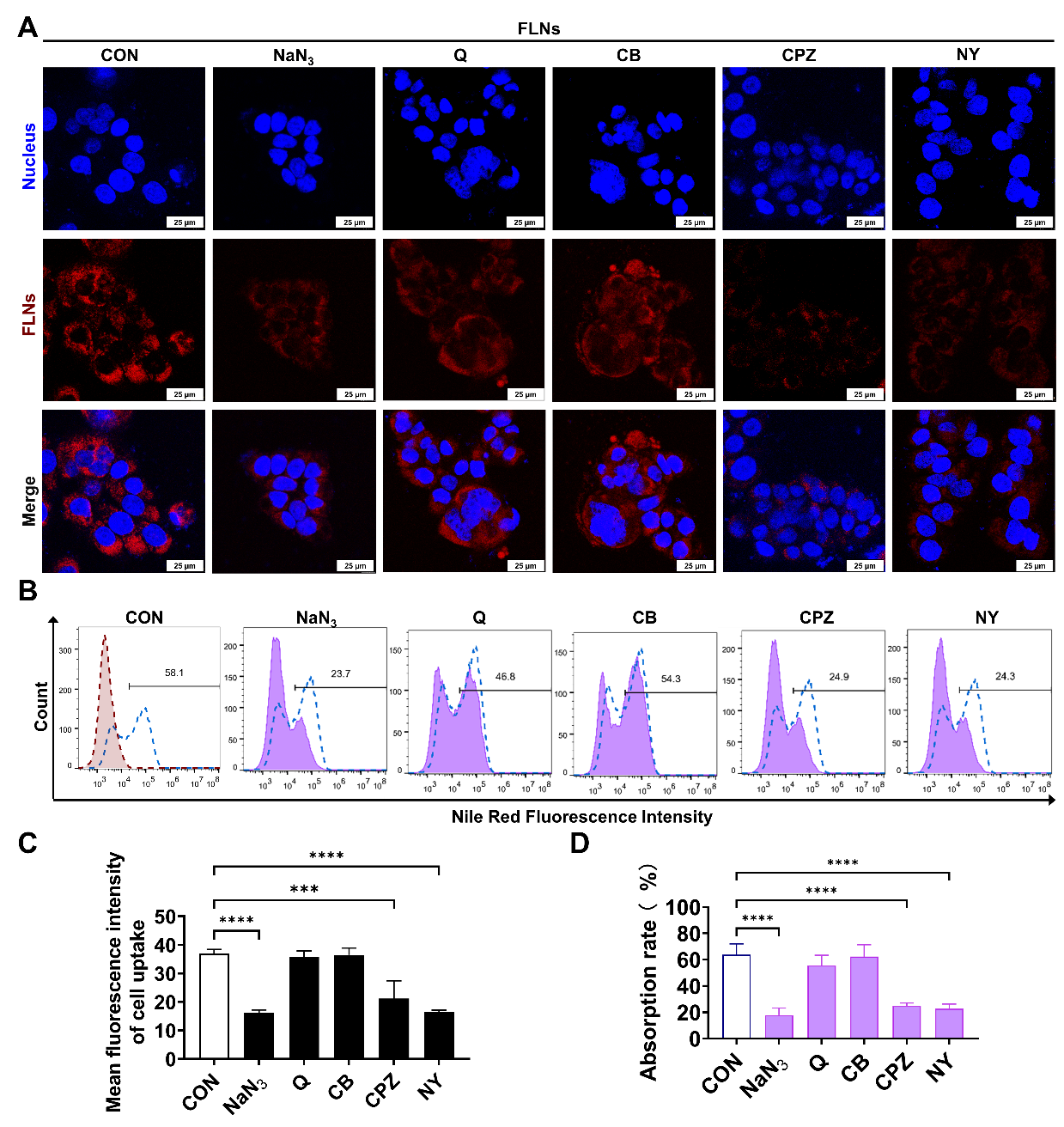


**Figure S4.** Internalization process of FLNs by cells. **A)**, The cellular uptake of FLNs observed by CLSM. Red fluorescence indicates Nile red-labelled FLNs. Blue fluorescence indicates DAPI-stained nuclei. Scale bars: 25 µm. **B)**, The endocytosis pathway of FLNs was analyzed by flow cytometry. The dashed line represents the absorption rate of cells treated with FLNs for 24 h. **C)**, Quantitative analysis of absorption dose of FLNs after being pretreated with different endocytic pathway inhibitors. **D)**, Quantitative analysis of the absorption rate of FLNs after being pretreated with different endocytic pathway inhibitors. Abbreviations: CON (Pretreatment without inhibitor), Q (quercetin), CB (cytochalasin B), CPZ (chlorpromazine), and NY (nystatin). **P* < 0.05; ***P* < 0.01; ****P* < 0.001 and *****P* < 0.0001. Data are presented as mean ± SD (n=3).

**
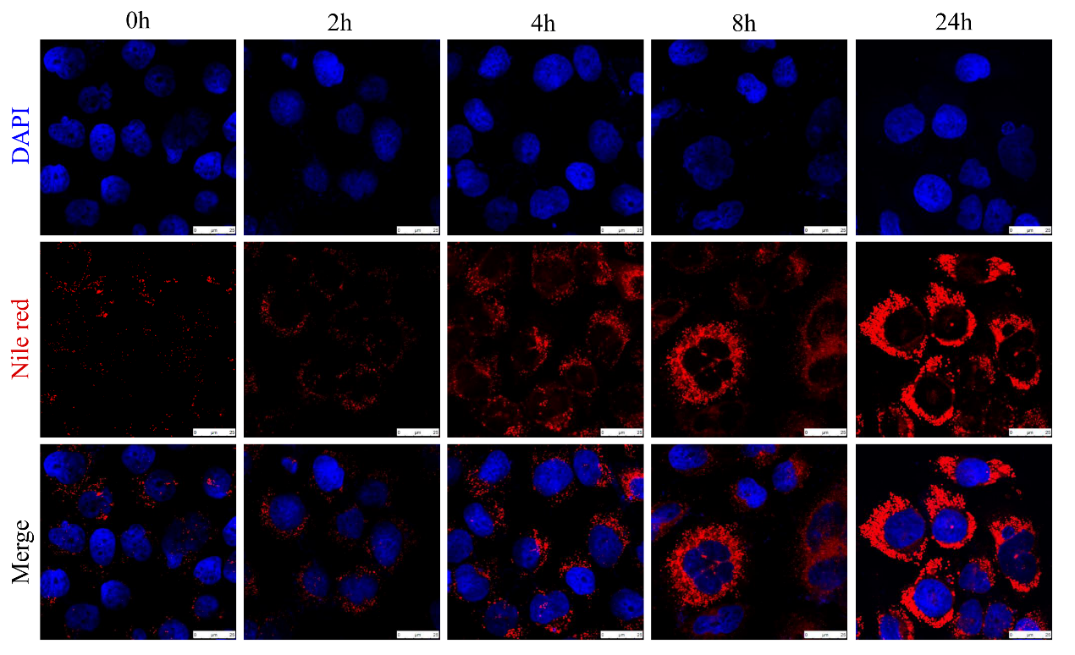
**

**Figure S5.** CLSM observation of LDs in Hela cells after FLNs treatment for different times, DAPI (blue) staining of cell nuclei, Nile red (red) staining of lipid droplets. Scale bars: 25 µm.


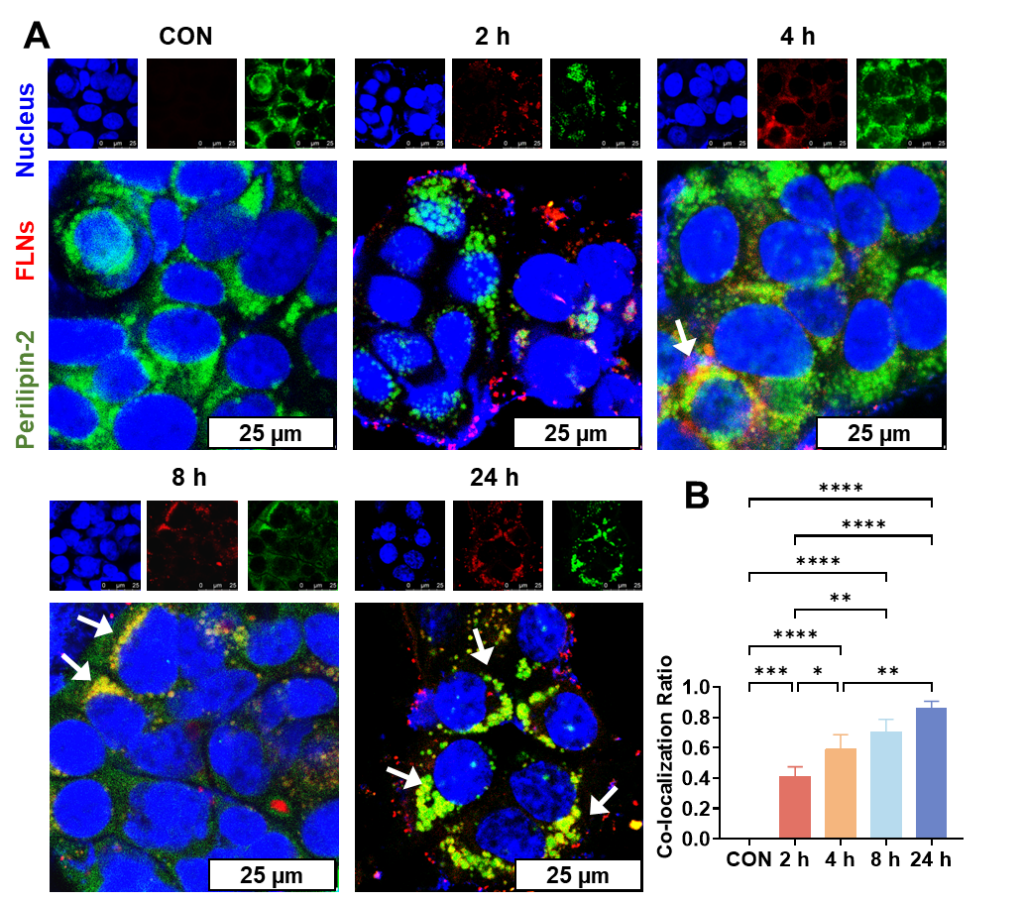


**Figure S6.** Detection of the colocalization of FLNs with Perilipin-2 proteins. A, CLSM detection of the colocalization of FLNs and Perilipin-2 protein; Blue: nucleus; Red: FLNs; Green: Perilipin-2. B, Quantitative analysis of FLNs and Perilipin-2 protein colocalization ratio. Data are presented as mean ± SD; n=3. **P* < 0.05; ***P* < 0.01; ****P* < 0.001 and *****P* < 0.0001. Data are presented as mean ± SD (n=3).


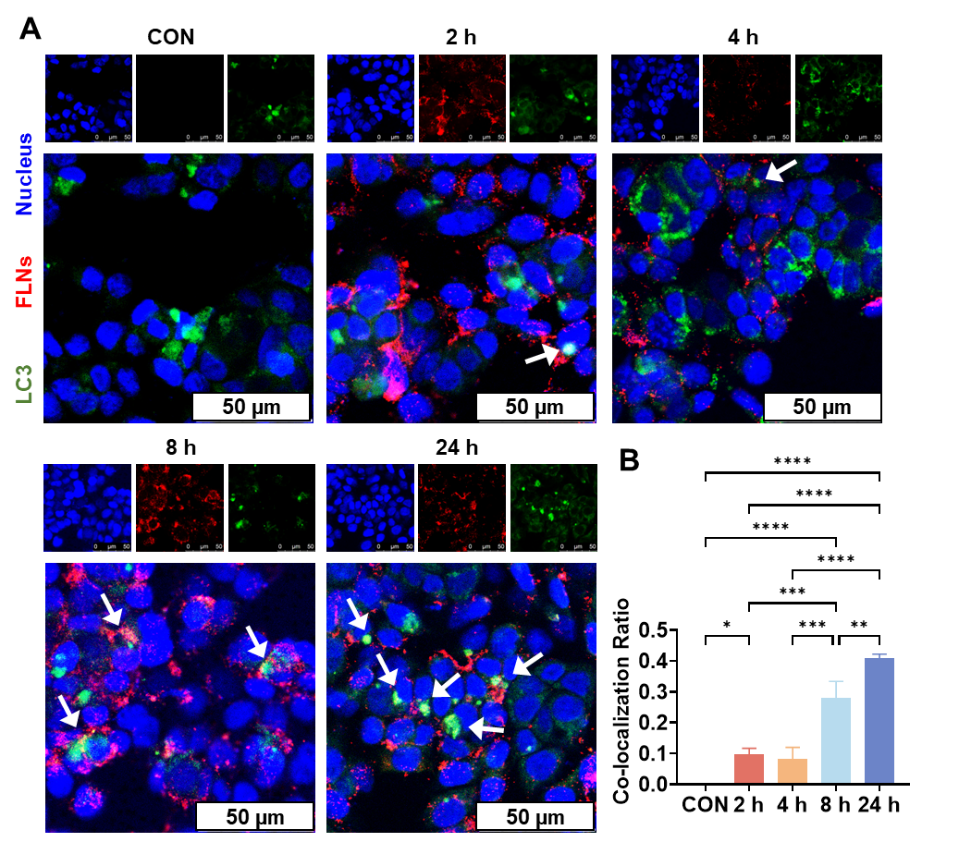


**Figure S7.** The colocalization of FLNs with LC3 proteins was detected. A, CLSM detection of the co-localization of FLNs and LC3 protein (White arrow); B, The colocalization ratio of FLNs and LC3 protein was quantitatively analyzed. Immunostained with an antibody against LC3 (green), DAPI (blue) stains nuclei, and Nile red (red) stains FLNs. White arrows indicate areas of colocalization. **P* < 0.05; ***P* < 0.01; ****P* < 0.001 and *****P* < 0.0001. Data are presented as mean ± SD (n=3).


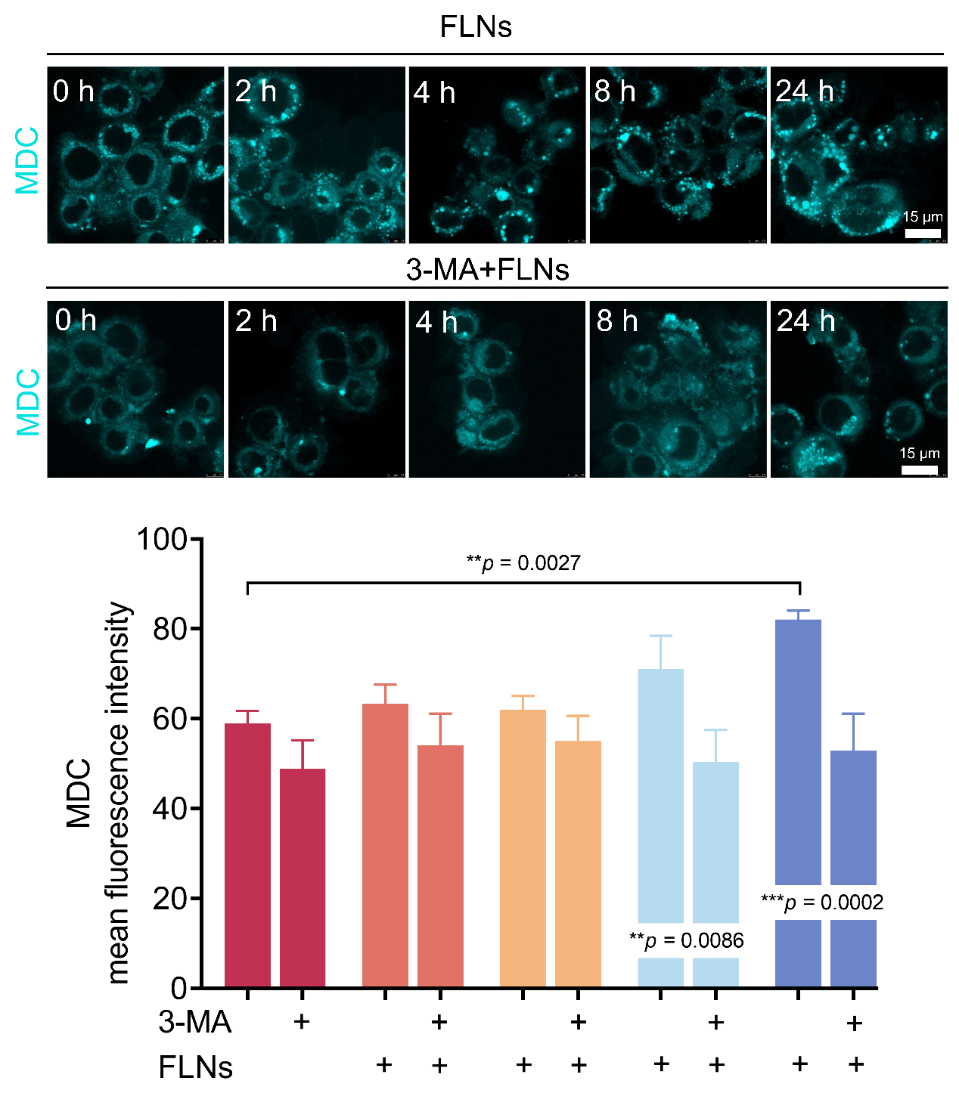


**Figure S8.** The effect of FLNs on autophagic flux in cells detected by MDC staining. Bright dots indicate autophagosomes. **P* < 0.05; ***P* < 0.01; ****P* < 0.001 and *****P* < 0.0001. Scale bars: 15 µm. Data are presented as mean ± SD (n=3).


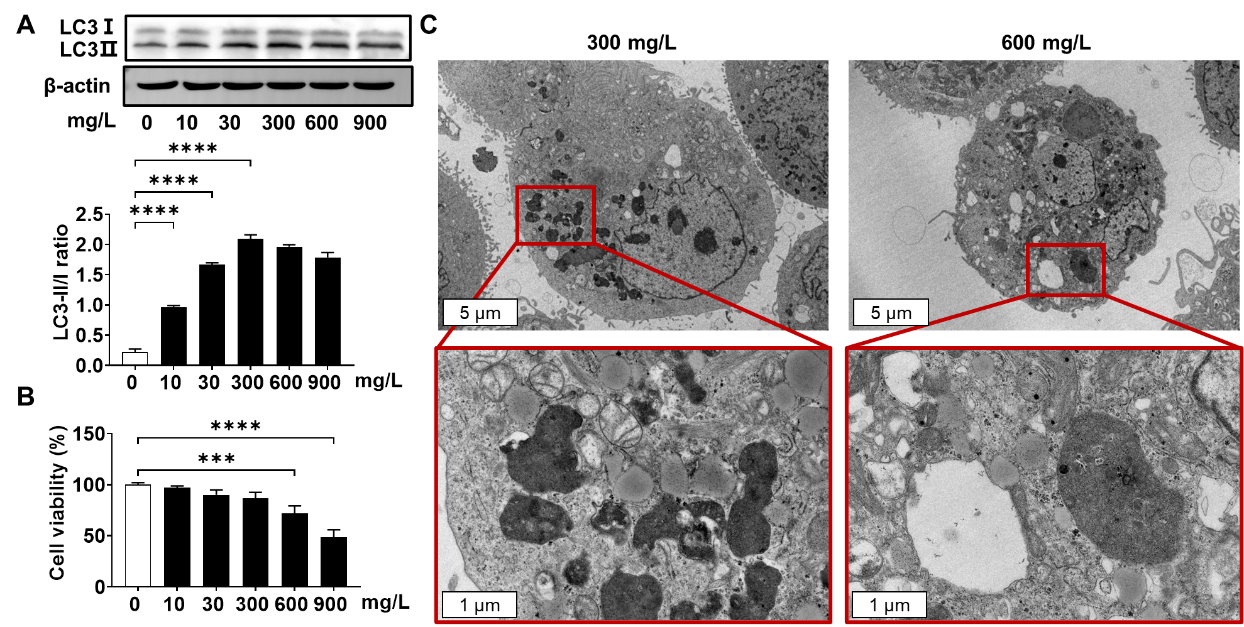


**Figure S9.** Effects of different concentrations of FLNs on autophagy levels of cells. **A)**, After treating cells with different concentrations (0, 10, 30, 300, 600, 900 µg/mL) of FLNs, the LC3Ⅱ/Ⅰ ratio was detected by Western blot. **B)**, The effects of different concentrations of FLNs on cell viability were analyzed. **C)**, TEM was used to observe the effect of different concentrations of FLNs on the autophagy level of cells; the image below is a larger view of the area in the red box. **P* < 0.05; ***P* < 0.01; ****P* < 0.001 and *****P* < 0.0001. Data are presented as mean ± SD (n=3).


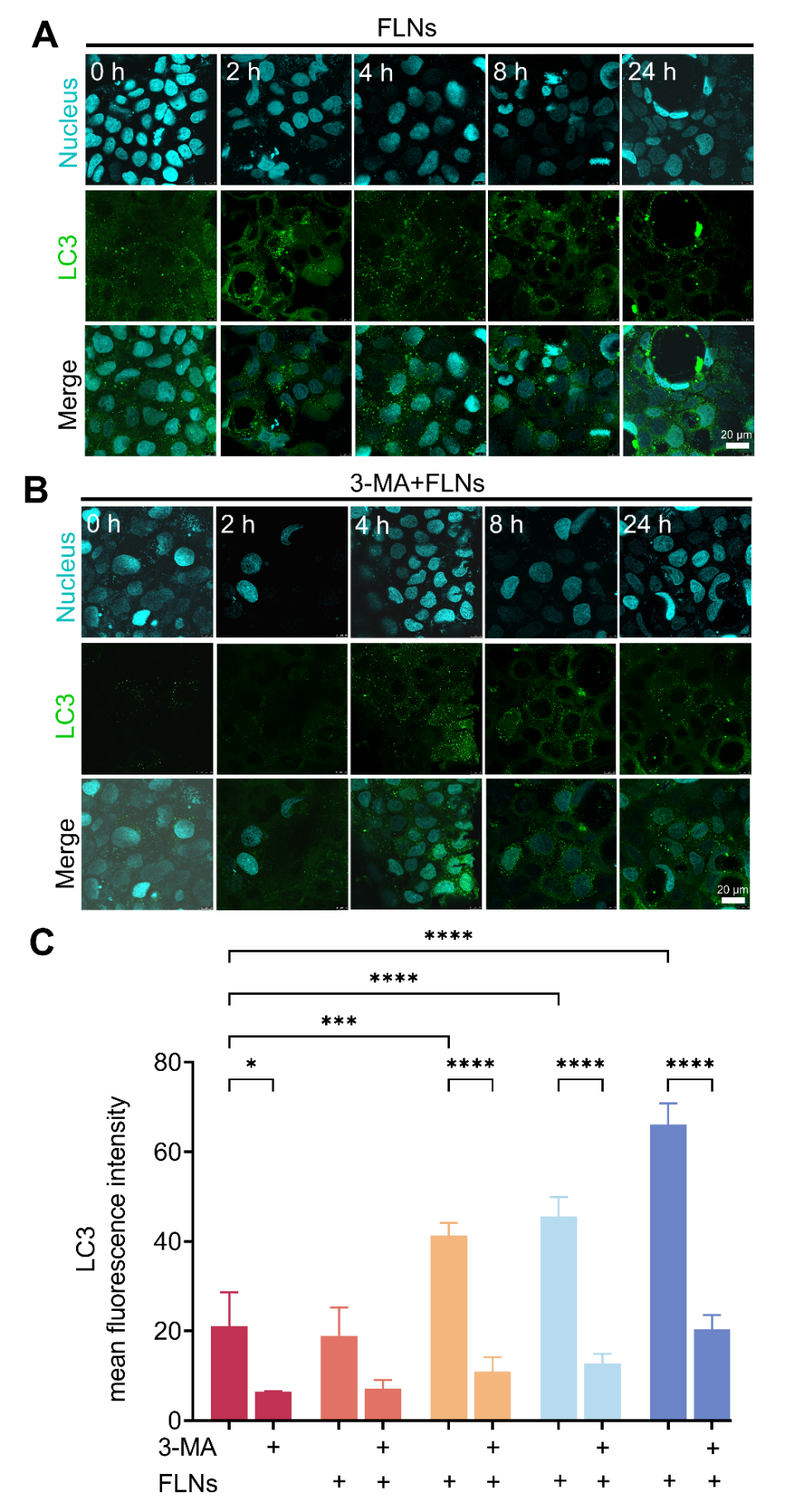


**Figure S10.** Detection of LC3 protein expression by immunofluorescence. **A)**, The LC3 level of cells was observed by CLSM after FLNs treatment. **B)**, CLSM observed the change of LC3 level in cells after FLNs treatment when autophagy was inhibited. **C)**, The LC3 level of cells was quantitatively analyzed. Scale bars: 20 µm. **P* < 0.05; ***P* < 0.01; ****P* < 0.001 and *****P* < 0.0001. Data are presented as mean ± SD (n=3).


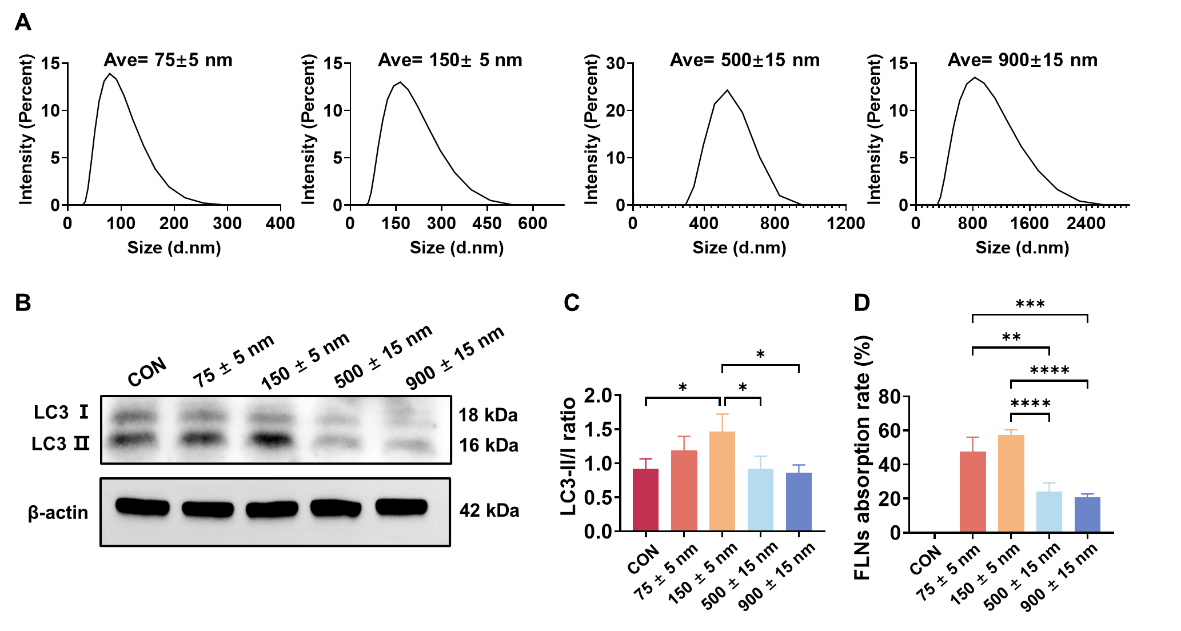


**Figure S11.** Effects of different sizes of FLNs on autophagic flux in cells. **A)**, Particle size detection of FLNs. **B)**, The effects of FLNs with different particle sizes on the autophagy level of cells were detected by Western blot. **C)**, The autophagy levels of cells treated by FLNs with different particle sizes were quantitatively analyzed. **D)**, The absorption rate of cells treated by FLNs with different particle sizes was quantitatively analyzed. **P* < 0.05; ***P* < 0.01; ****P* < 0.001 and *****P* < 0.0001. Data are presented as mean ± SD (n=3).


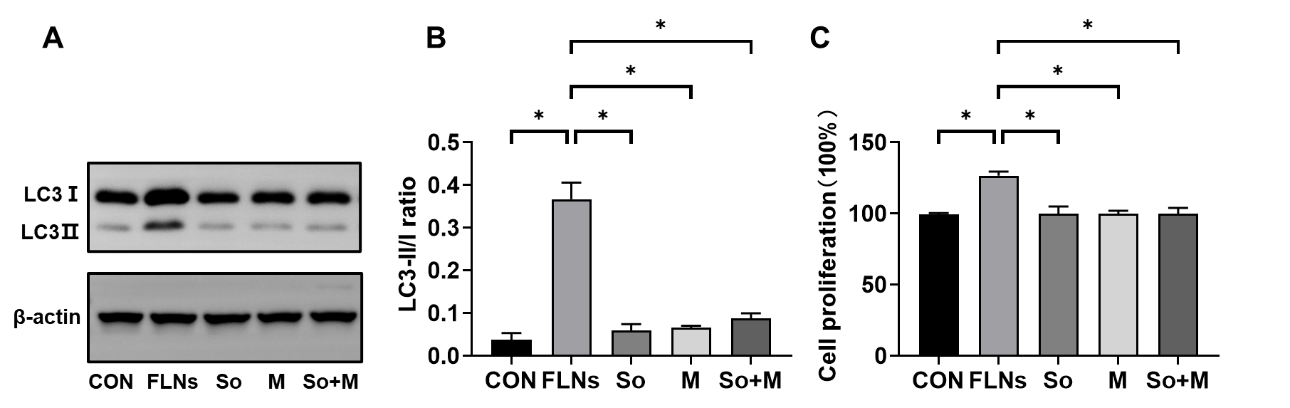


**Figure S12.** Effects of the components of FLNs on autophagy and cell proliferation. **A)**, The effects of FLNs components on autophagy were investigated using Western blot analysis. **B)**, The effects of the individual components of FLNs on autophagy were quantitatively analyzed. **C)**, Assessment of the Effects of FLNs components on cell proliferation using the Alamar Blue assay. Data are presented as mean ± SD; n=3 independent experiments. Abbreviations: CON (Control group), FLNs (Food-grade lipid nanoparticles), So (Soybean oil), M (Monoglyceride). **P* < 0.05; ***P* < 0.01; ****P* < 0.001 and *****P* < 0.0001. Data are presented as mean ± SD (n=3).


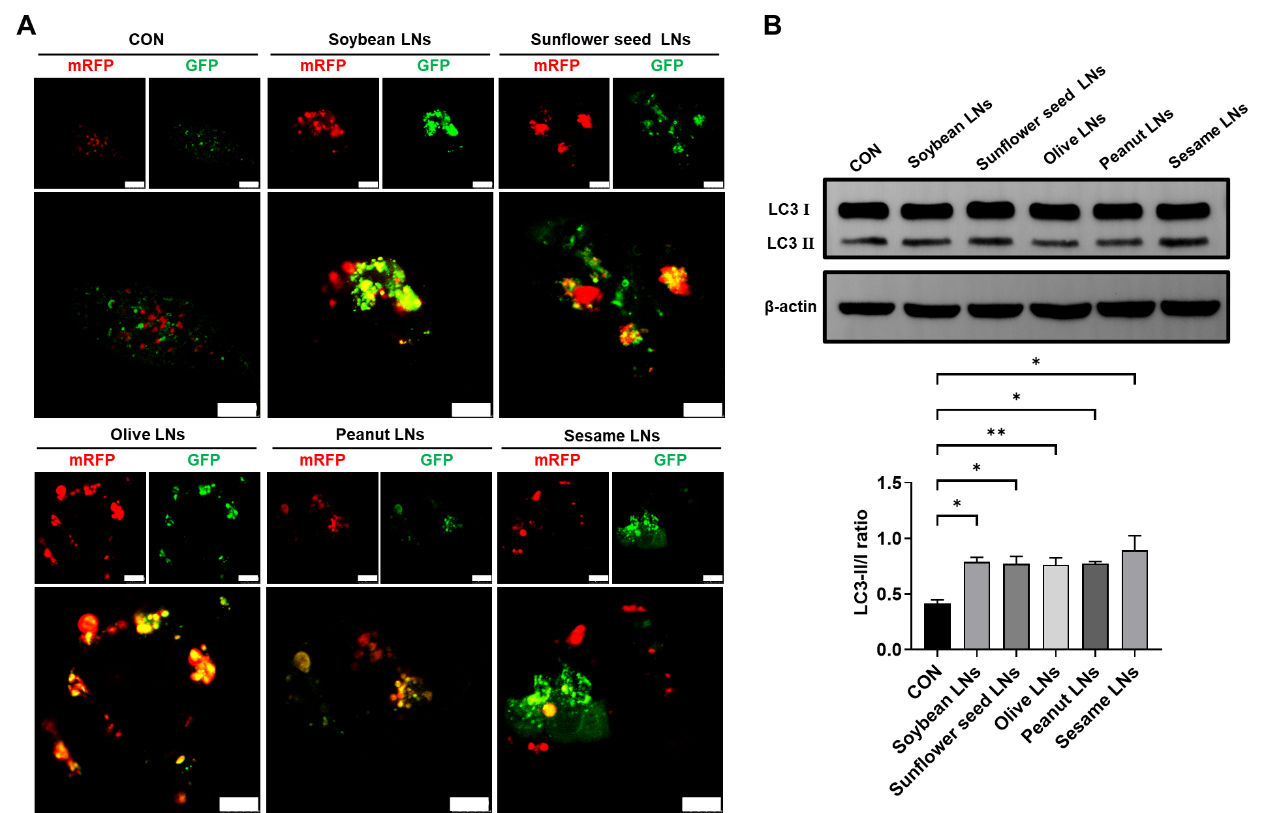


**Figure S13.** Effects of different lipid nanoparticles on autophagy levels. **A)**, Analysis of dynamic autophagy flux using transfected mRFP-GFP-LC3 caco-2 cell line. **B)**, Western blot analyses of LC3Ⅱ/Ⅰ ratio. Abbreviations: CON (Control group), LNs (lipid nanoparticles). Scale bars: 10 µm. **P* < 0.05; ***P* < 0.01; ****P* < 0.001 and *****P* < 0.0001. Data are presented as mean ± SD (n=3).


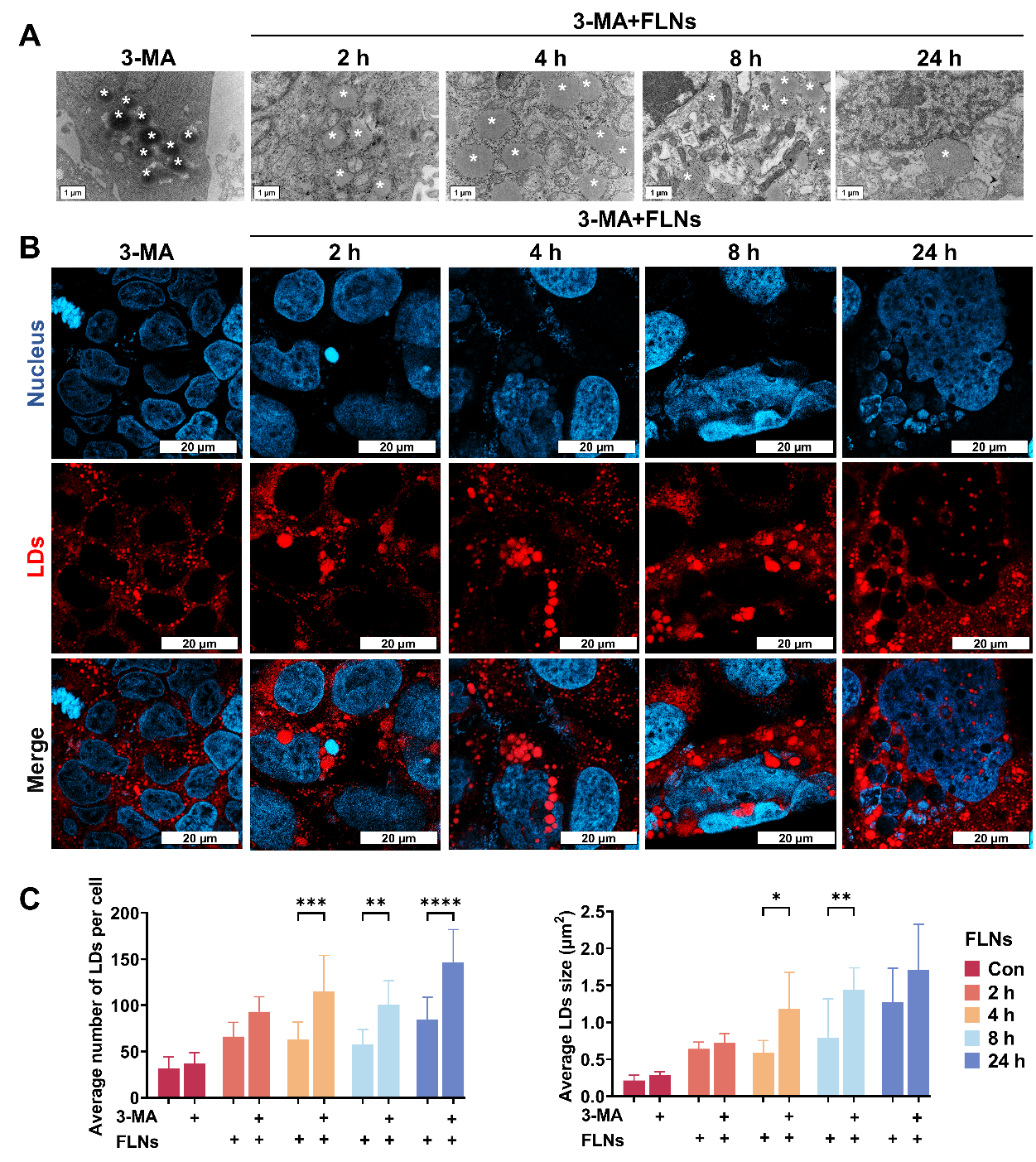


**Figure S14.** After autophagy was inhibited, the effect of FLNs on intracellular lipid droplets was observed by TEM and CLSM. **A)**, TEM observed lipid droplets (LDs) inside cells; Star (*) is used to signify the LDs; Scale bars: 1 µm. **B)**, CLSM observed lipid droplets (LDs) inside cells. DAPI (blue) stains nuclei and Nile red (red) stains LDs; Scale bars: 20 µm. **C)**, The size and number of LDs were quantitatively analyzed. **P* < 0.05; ***P* < 0.01; ****P* < 0.001 and *****P* < 0.0001. Data are presented as mean ± SD (n=3).


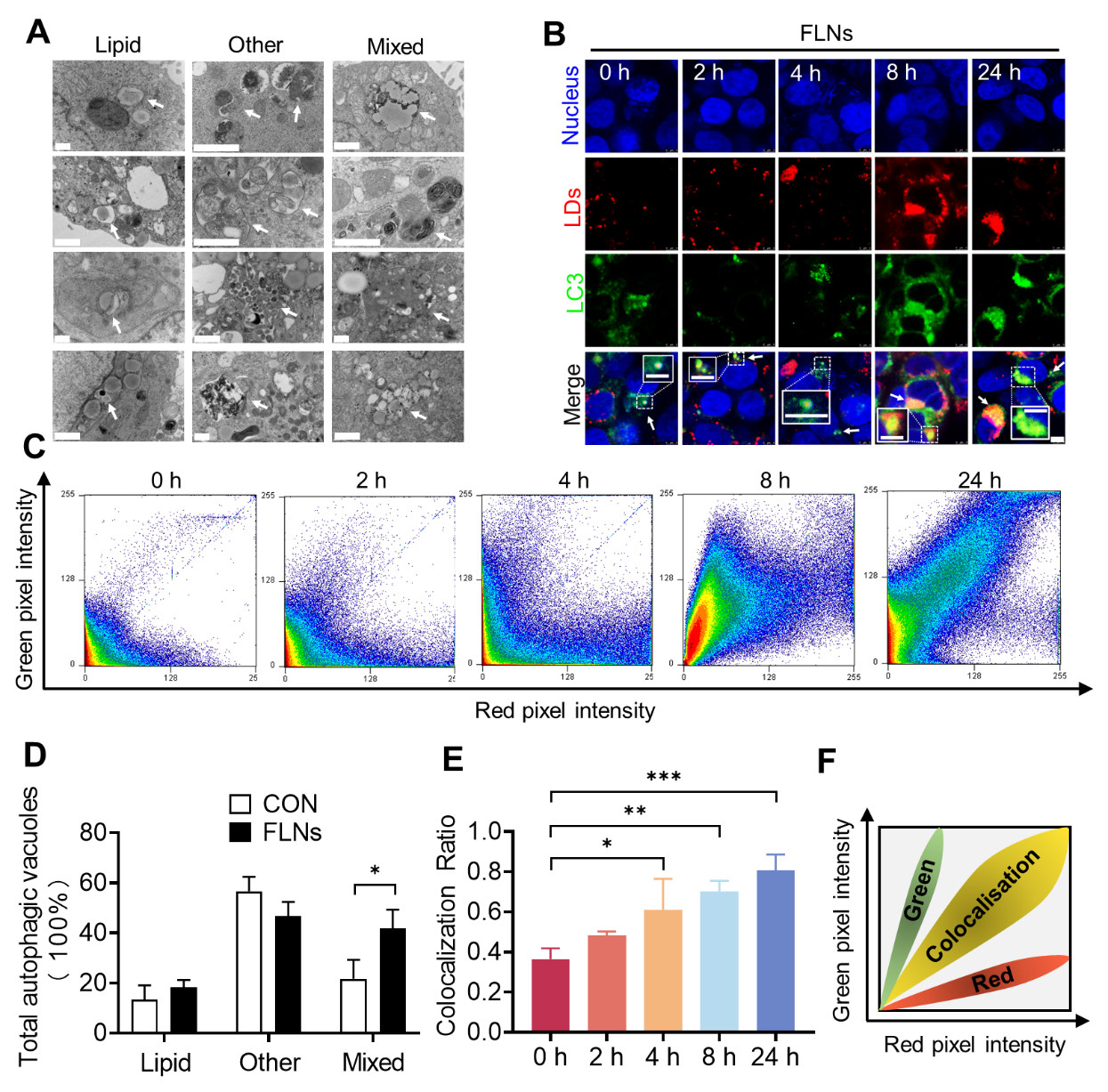


**Figure S15.** Autophagy regulates LDs metabolism. **A)**, TEM observation of autophagosomes in cells that contain only lipids droplets (LDs), other substances (Other), or LDs and other organelles mixed substance (Mixed); the white arrows show a partial enlarged image. Scale bars, 1 µm. **B)**, Immunostained with an antibody against LC3(green), DAPI (blue) stains nuclei and Nile red (red) stains LDs; white boxes indicate areas of colocalization; the white box shows a partial enlarged image. Scale bars: 5 µm; magnification scale bars: 5 µm. **C)**, Co-localization scatter plot analysis. **D)**, Percentage of autophagosomes in cells that contain only lipids droplets (LDs), other substances (Other), or LDs and other organelles mixed substance (Mixed). **E)**, Quantitative analysis of AP (autophagosomes) and LDs colocalization ratio. **F)**, Schematic of assay for AP (autophagosomes) and LDs colocalizations. **P* < 0.05; ***P* < 0.01; ****P* < 0.001 and *****P* < 0.0001. Data are presented as mean ± SD (n=3).


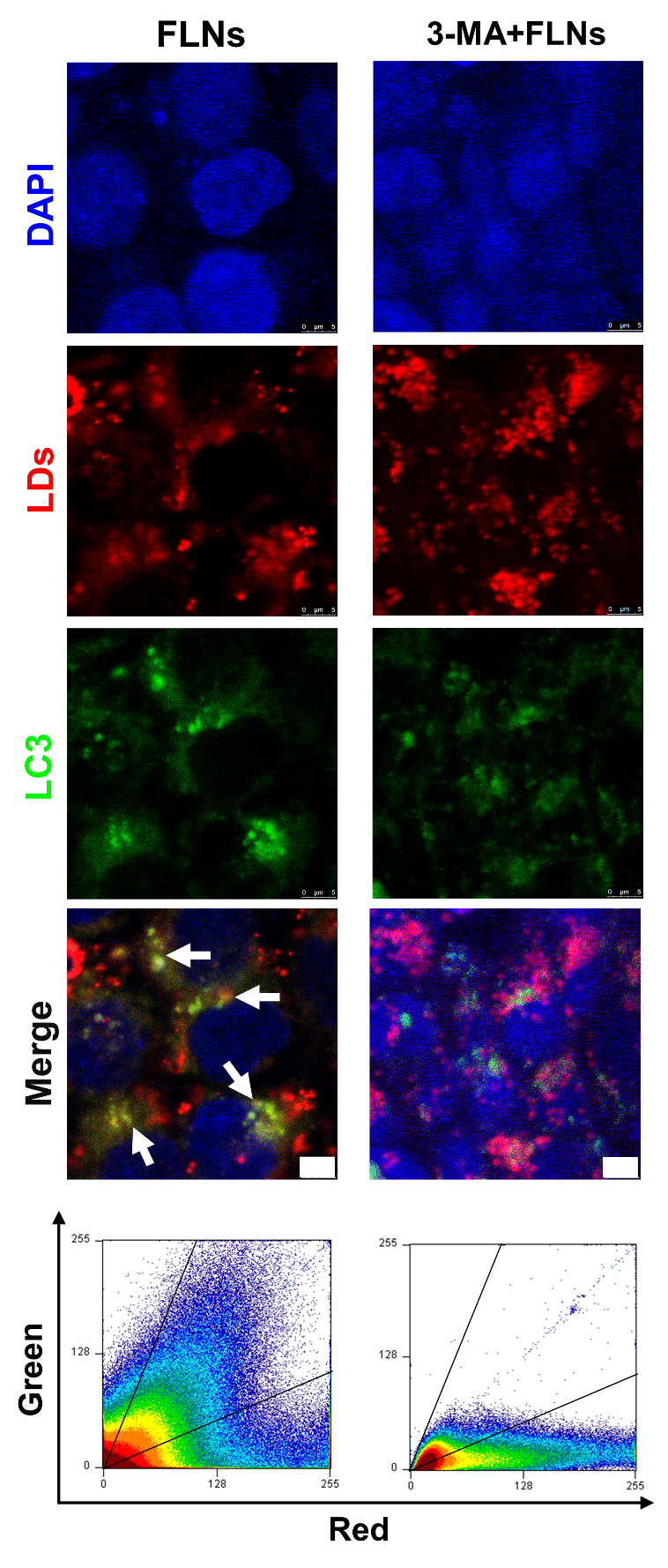


**Figure S16.** When autophagy was inhibited, CLSM detected the colocalization of lipid droplets and autophagosomes after FLNs treatment. Immunostained with an antibody against LC3(green), DAPI (blue) stains nuclei, and Nile red (red) stains lipid droplets; white arrows indicate areas of colocalization. FLNs: cells were treated with FLNs for 24 h. 3-MA+FLNs: After pretreatment with 3-MA, cells were treated with FLNs for 24 h. Scale bars: 5 µm. Data are presented as mean ± SD (n=3).


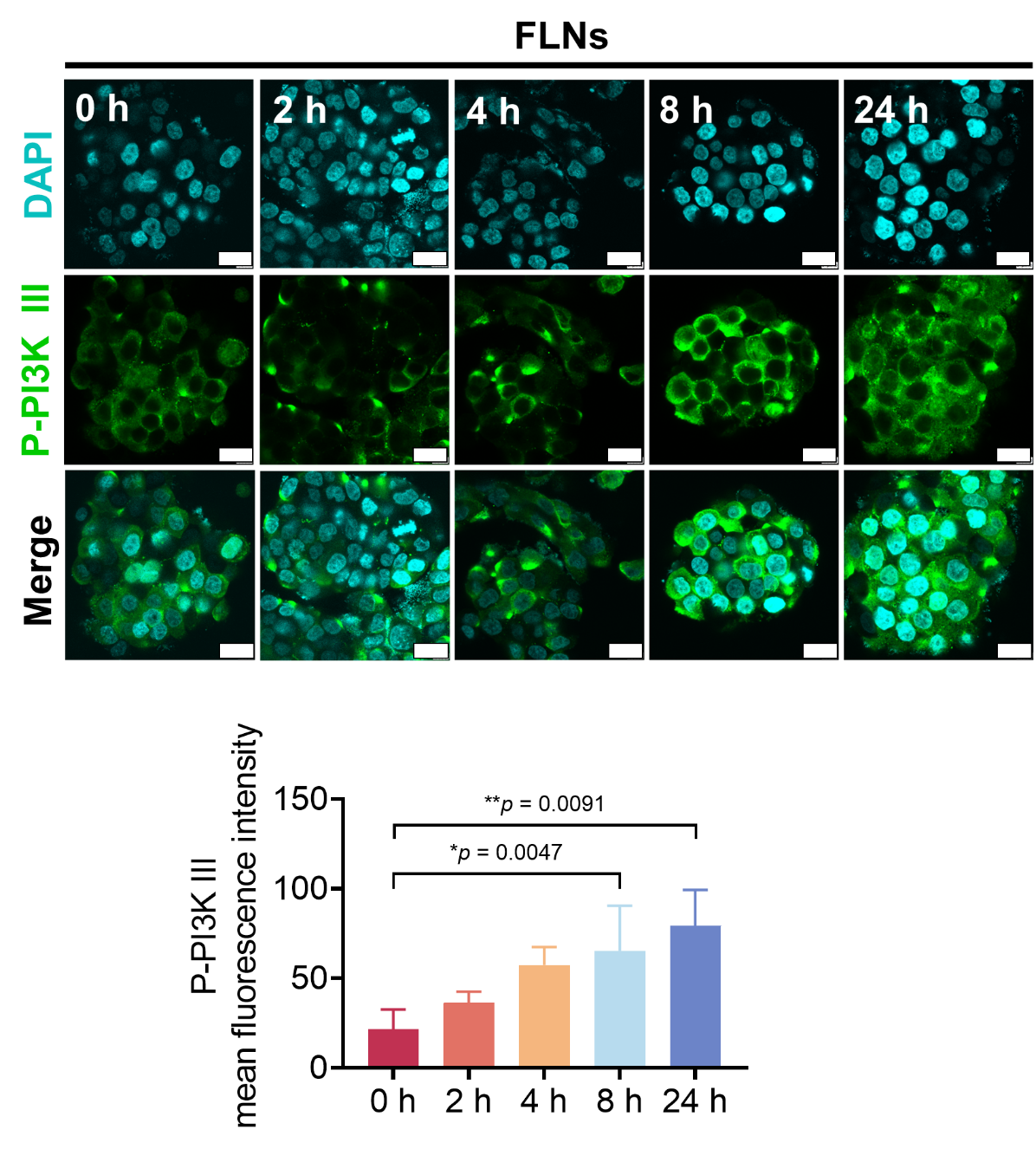


**Figure S17.** CLSM detection of P-Class III PI3K protein expression in cells after FLNs treatment; Immunostained with an antibody against Class III PI3K (green), DAPI (blue) staining of cell nuclei. Scale bars: 20 µm; **P* < 0.05; ***P* < 0.01; ****P* < 0.001 and *****P* < 0.0001. Data are presented as mean ± SD (n=3).


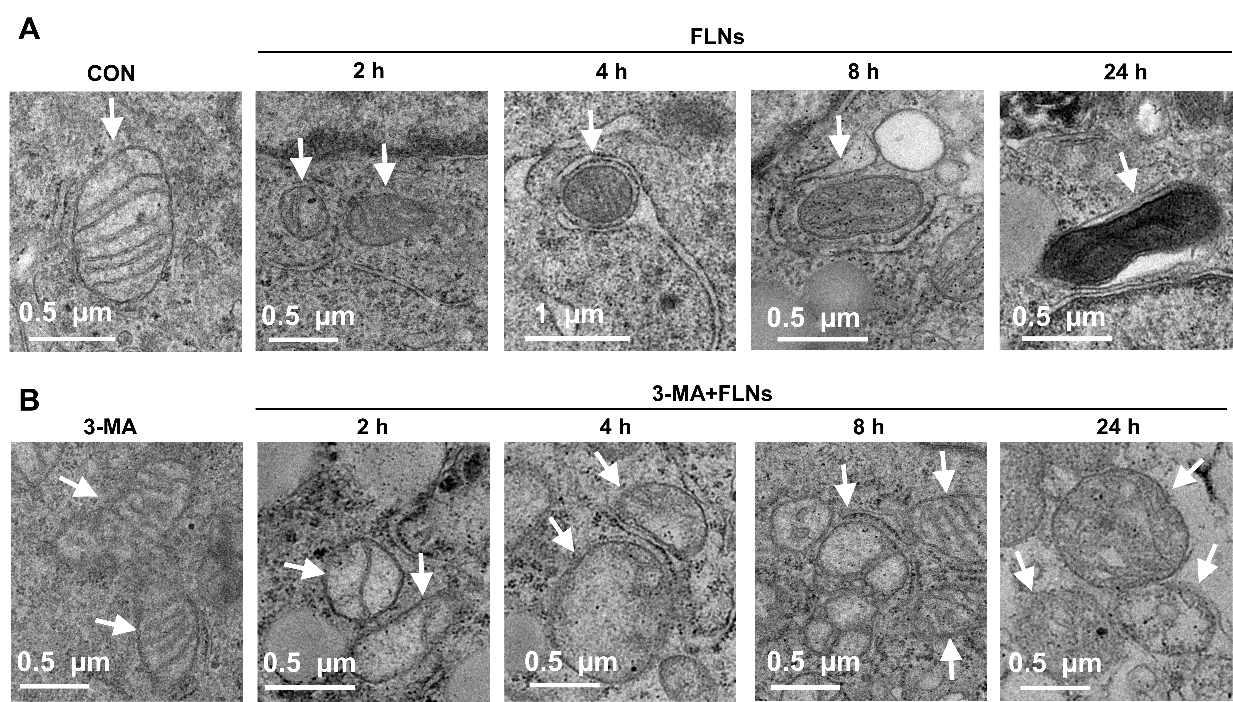


**Figure S18.** TEM observation of mitochondrial morphology, the white arrows indicate mitochondria. **A)**, After FLNs treatment of cells, mitochondrial morphology was observed at different time periods. **B)**, Under the premise of inhibiting autophagy, the mitochondrial morphology of cells was observed at different time periods after FLNs treatment. The white arrows indicate mitochondria. n=3.


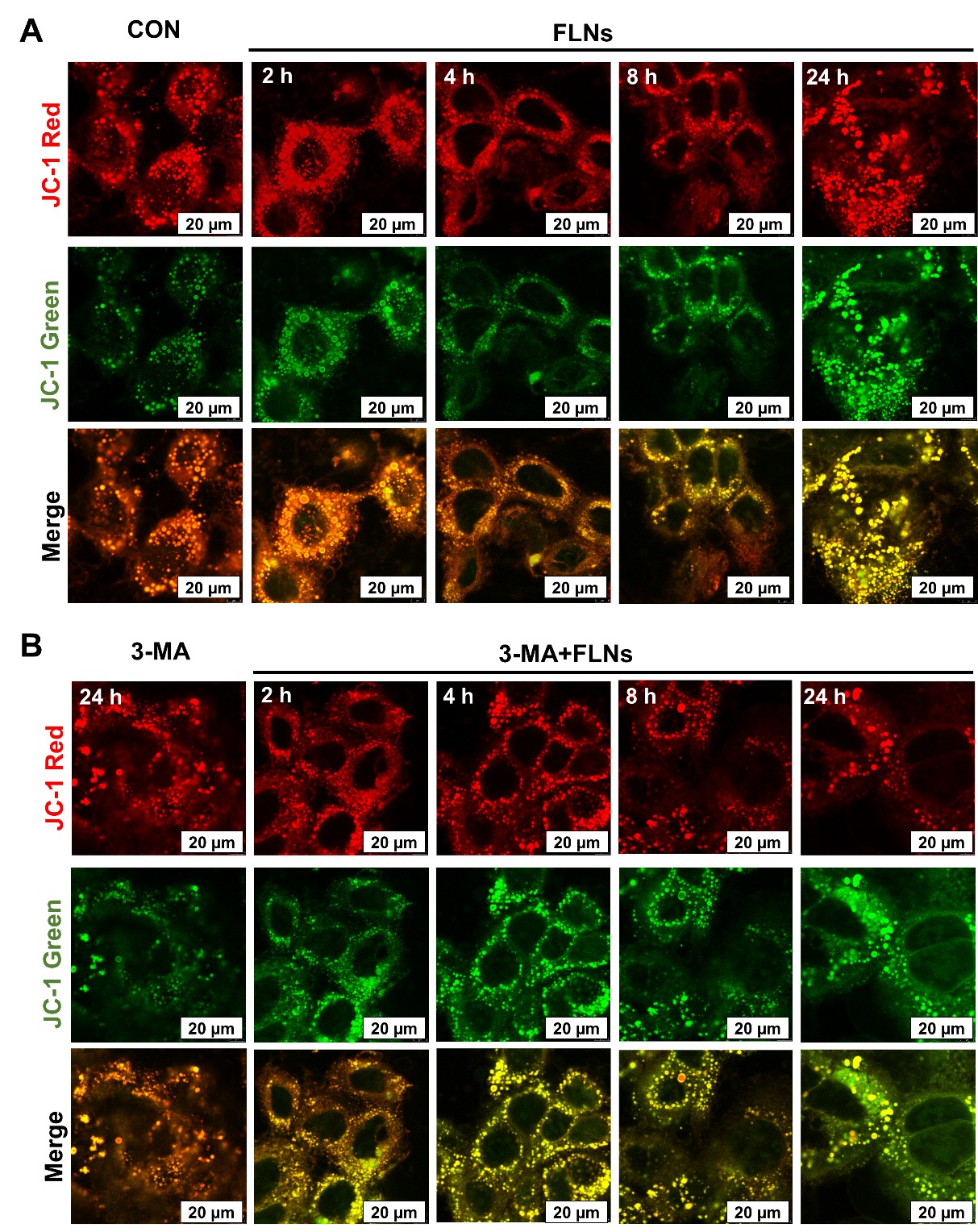


**Figure S19.** Effect of FLNs on mitochondrial membrane potential with and without inhibition of autophagy. **A)**, CLSM observation of mitochondrial membrane potential. **B)**, Quantitative analysis of mitochondrial membrane potential. Green: JC-1 monomer; Red: JC-1 aggregates. 3-MA: 3-MA treatment for 24 h. 3-MA+ FLNs: 3-MA pretreatment for 1h and then FLNs treatment for different time. n=3.


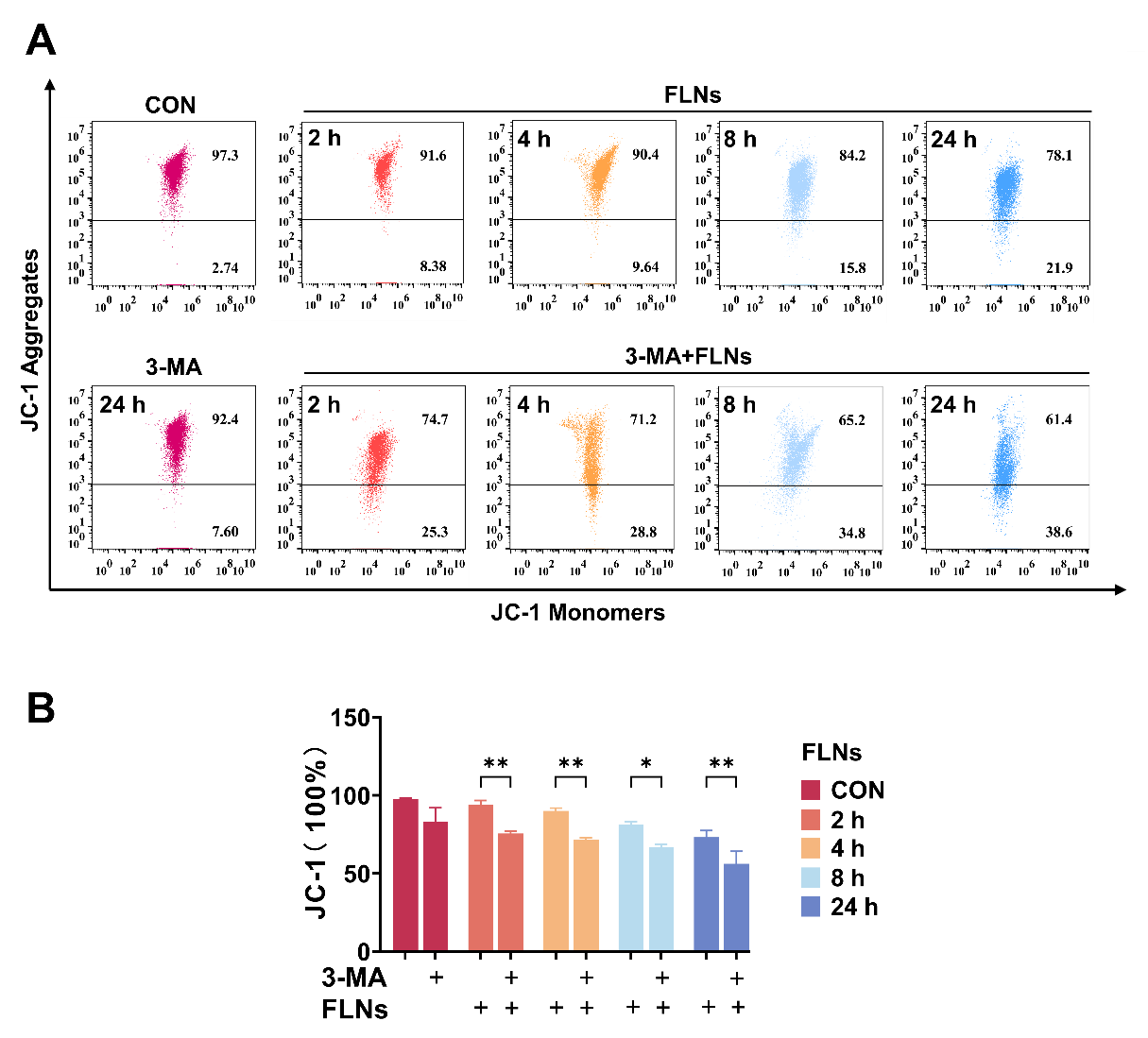


**Figure S20.** Effect of FLNs on mitochondrial membrane potential with and without inhibition of autophagy. **A)**, Flow cytometer detection of mitochondrial membrane potential. **B)**, Quantitative analysis of mitochondrial membrane potential. **P* < 0.05; ***P* < 0.01; ****P* < 0.001 and *****P* < 0.0001. Data are presented as mean ± SD (n=3).


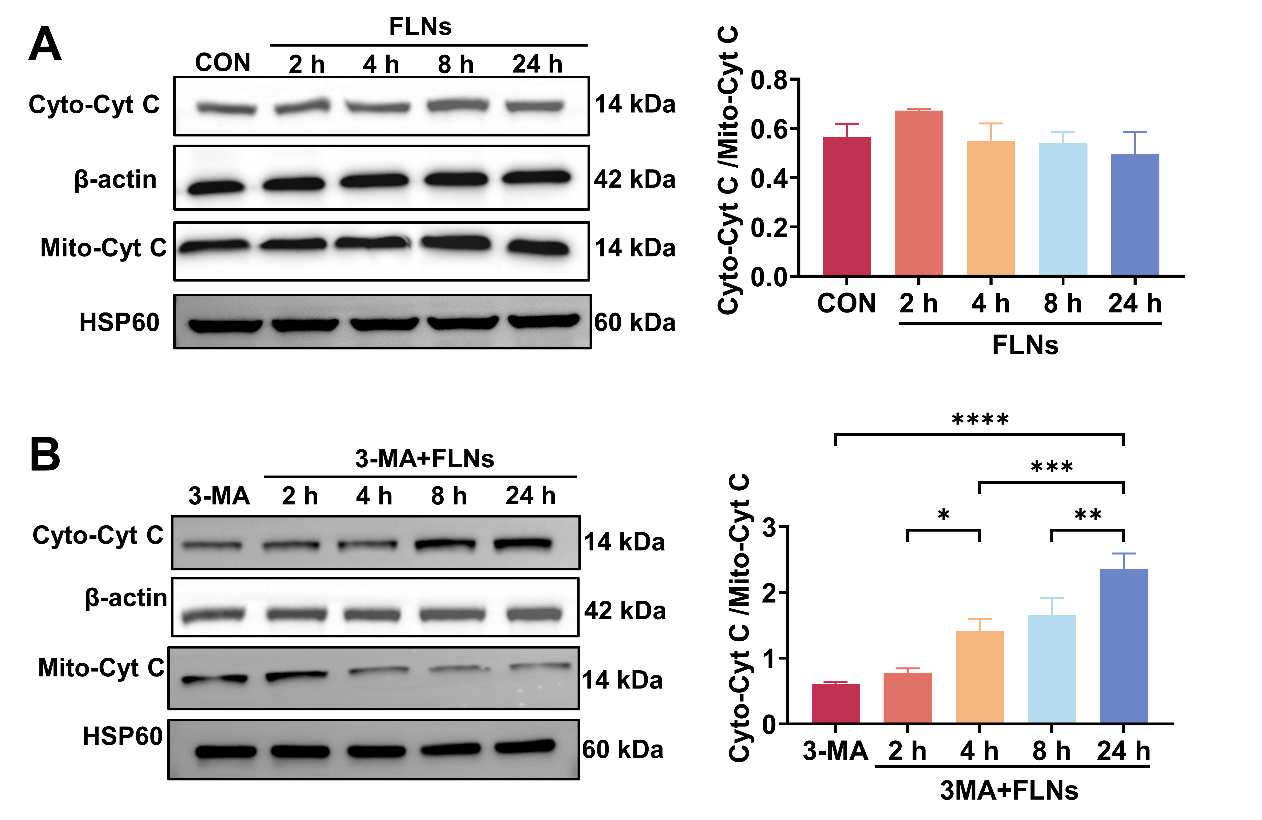


**Figure S21.** Effect of FLNs on mitochondrial Cyt-c release with and without inhibition of autophagy. **A)**, Effect of FLNs on mitochondrial Cyt-c release. **B)**, Effect of FLNs on mitochondrial Cyt-c release when autophagy is inhibited. **P* < 0.05; ***P* < 0.01; ****P* < 0.001 and *****P* < 0.0001. Data are presented as mean ± SD (n=3).

**
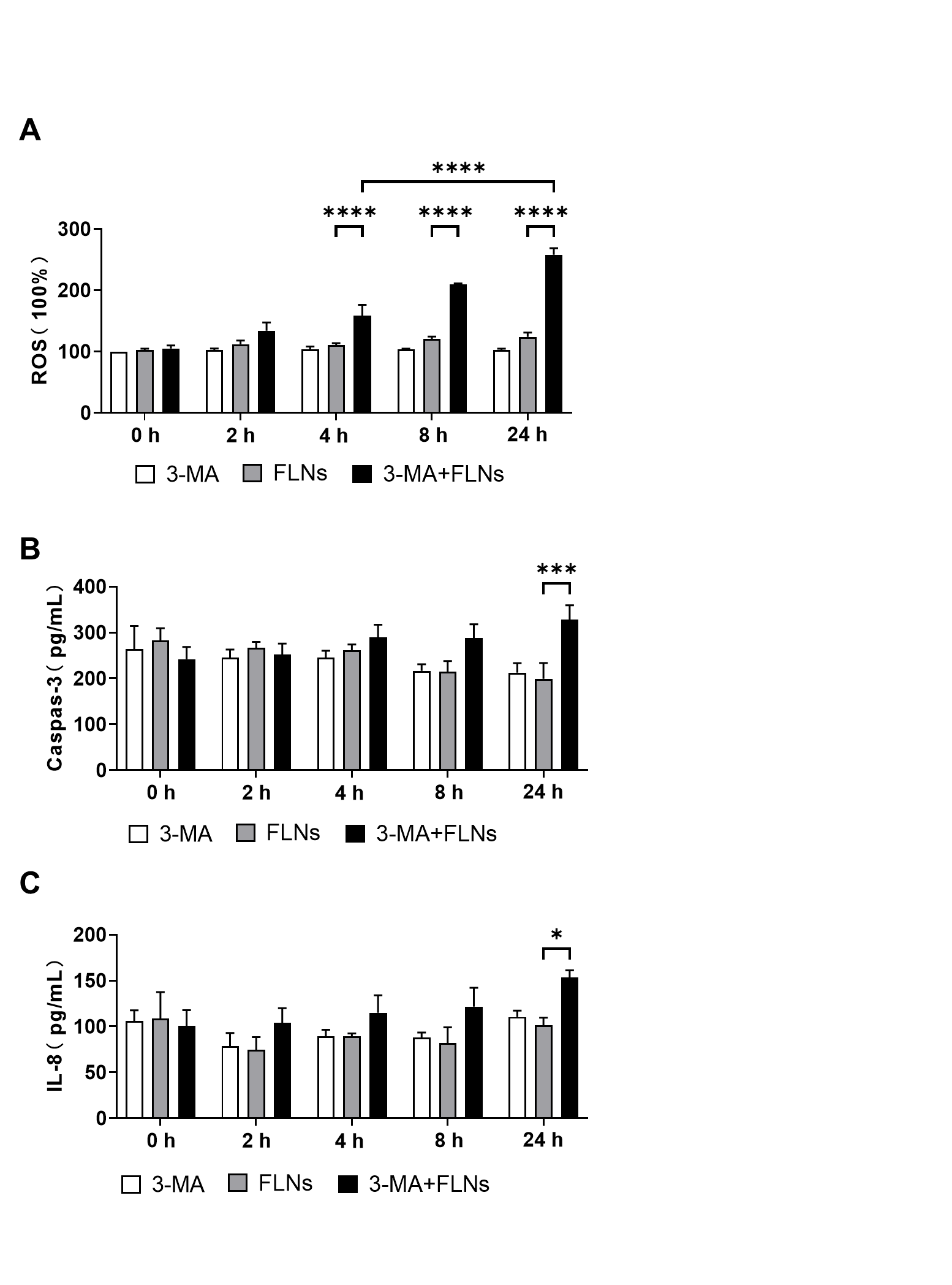
**

**Figure S22.** Cells were pretreated with or without 3-MA and then treated with FLNs, intracellular **A**) ROS, **B**) Caspase-3 and **C**) IL-8 changes were detected. **P* < 0.05; ***P* < 0.01; ****P* < 0.001 and *****P* < 0.0001. Data are presented as mean ± SD (n=3).

**
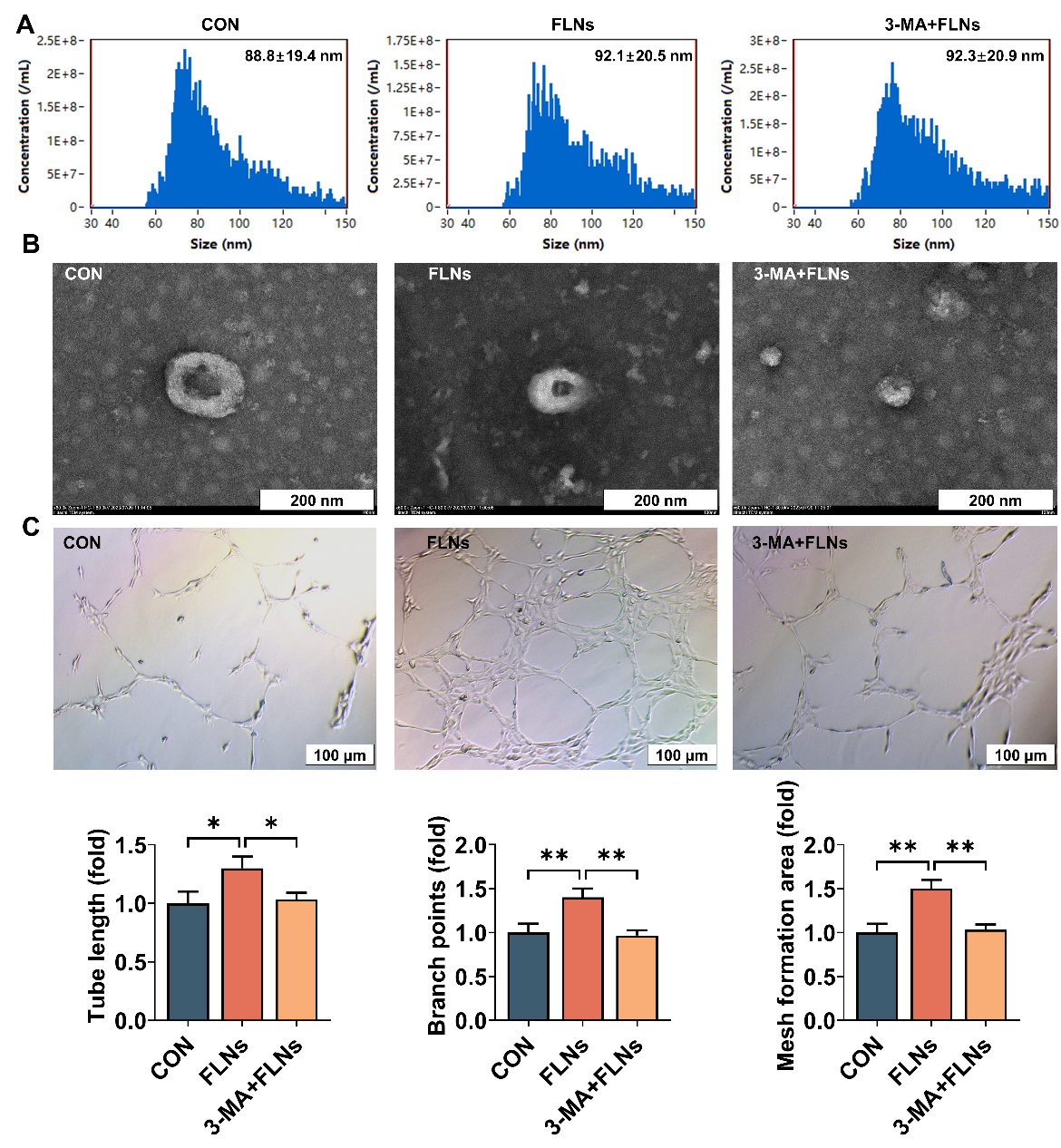
**

**Figure S23.** FLNs-treated cells extracellular vesicles induce angiogenesis. **A)**, The particle size of cell-generated vesicles treated with and without FLNs treatment. **B)**, The morphology of cell-generated vesicles with and without LNs treatment by transmission electron microscope. **C)**, Effects of FLNs-treated cells extracellular vesicles on angiogenesis in HUVEC cells by Optical microscope. Data are presented as mean ± SD (n=3).


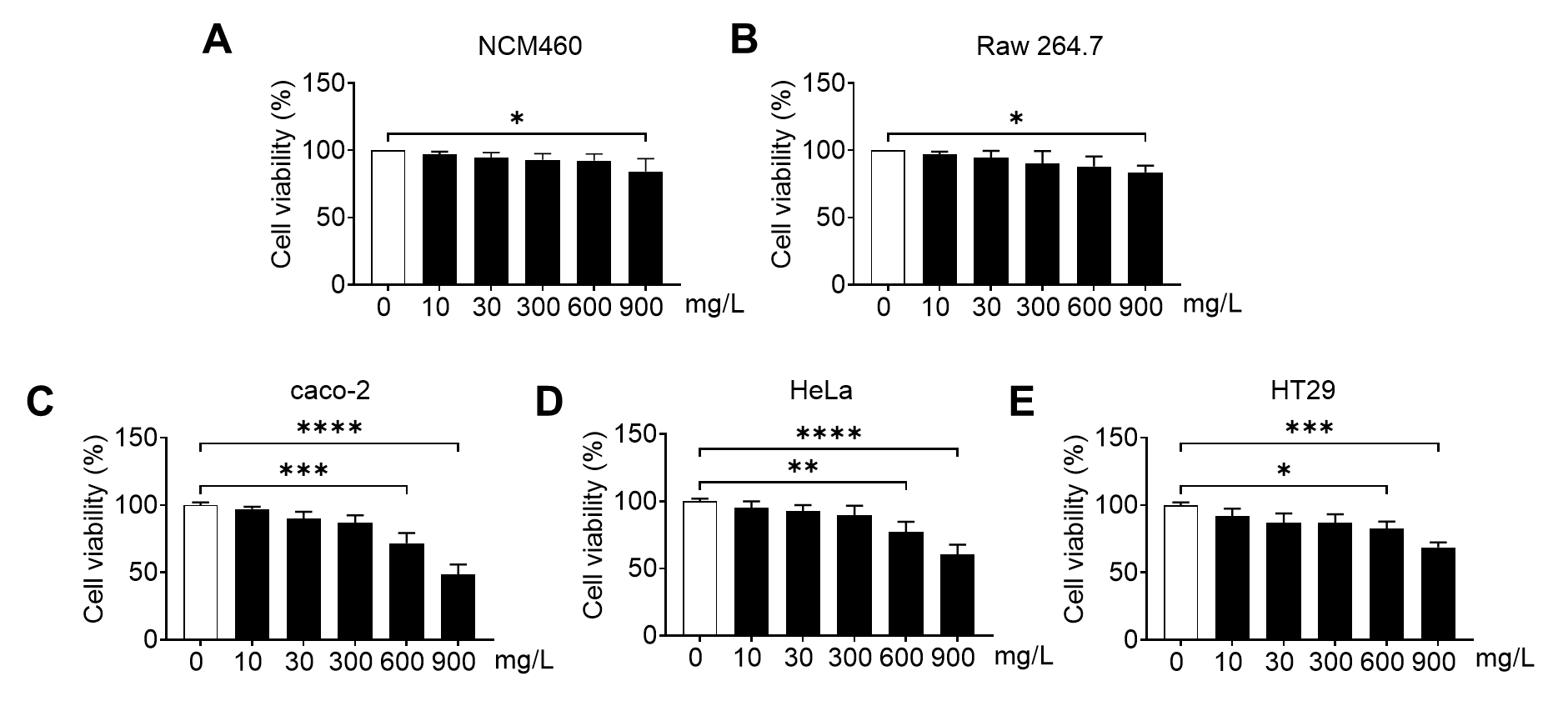


**Figure S24.** Effects of different concentrations of FLNs on viability rate of **A**) NCM460, **B**) RAW264.7, **C**) Caco-2, **D**) HeLa and **E**) HT29 cells. **P* < 0.05; ***P* < 0.01; ****P* < 0.001 and *****P* < 0.0001. Data are presented as mean ± SD (n=3).


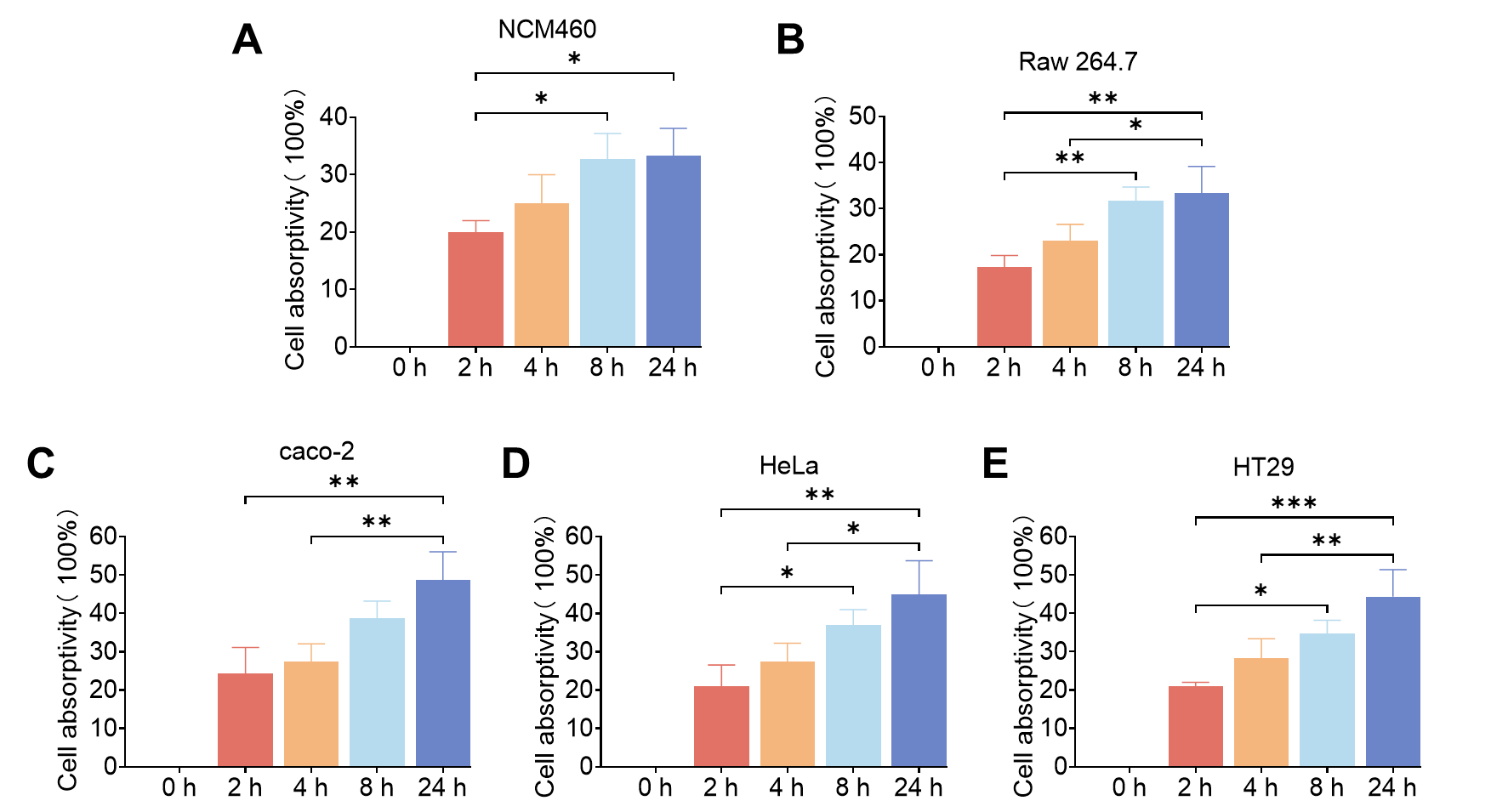


**Figure S25.** Effects of FLNs treatment time on viability rate of **A**) NCM460, **B**) RAW264.7, **C**) Caco-2, **D**) HeLa and **E**) HT29 cells. **P* < 0.05; ***P* < 0.01; ****P* < 0.001 and *****P* < 0.0001. Data are presented as mean ± SD (n=3).


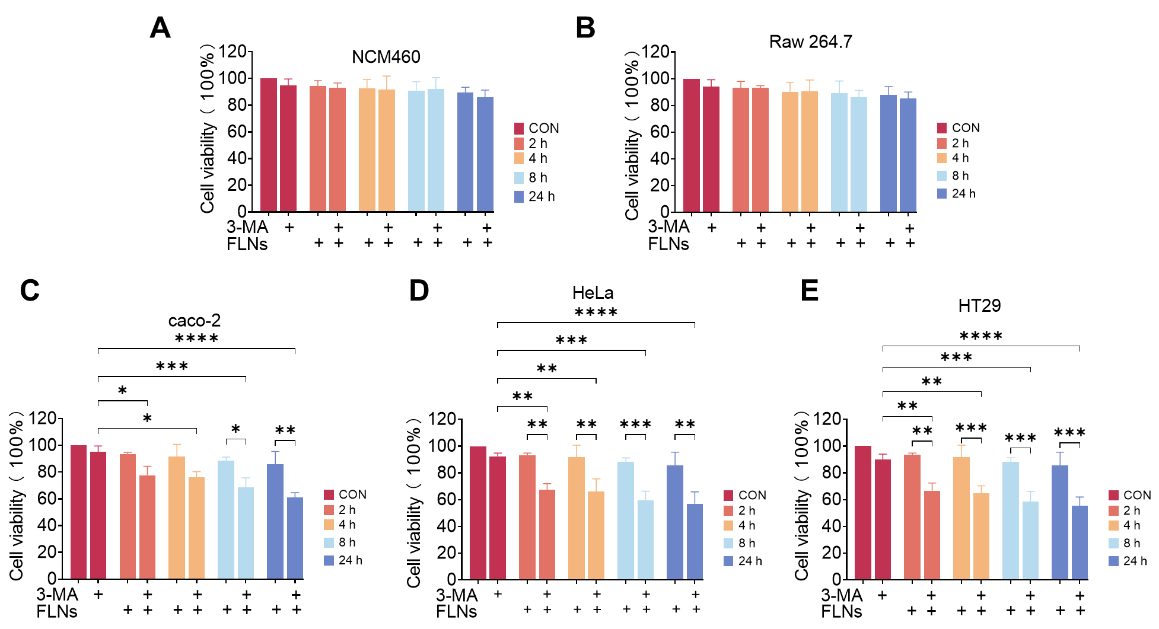


**Figure S26.** Effects of FLNs alone or in combination with 3-MA on viability rate of **A**) NCM460, **B**) RAW264.7, **C**) Caco-2, **D**) HeLa and **E**) HT29 cells. **P* < 0.05; ***P* < 0.01; ****P* < 0.001 and *****P* < 0.0001. Data are presented as mean ± SD (n=3).

**
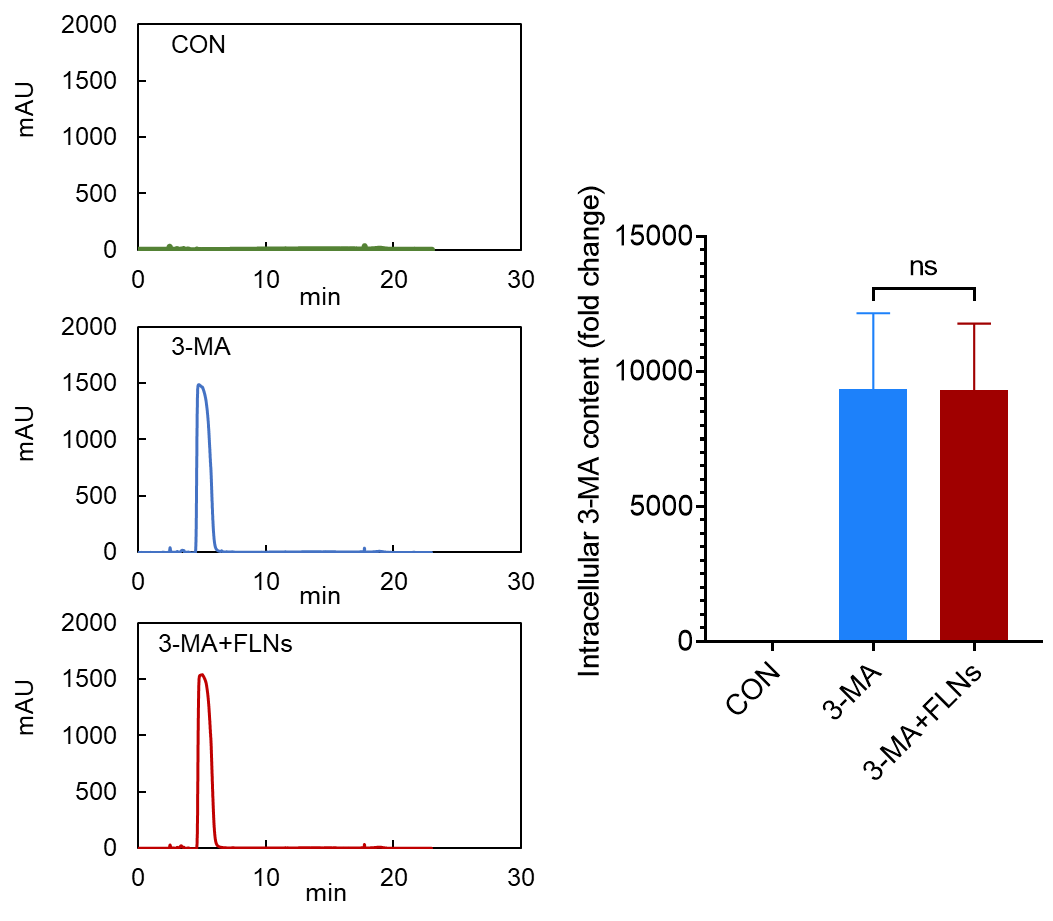
**

**Figure S27.** Liquid chromatography was used to detect the effect of FLNs on the absorption of 3-MA by cells. Data are presented as mean ± SD; n=3 independent experiments.

**
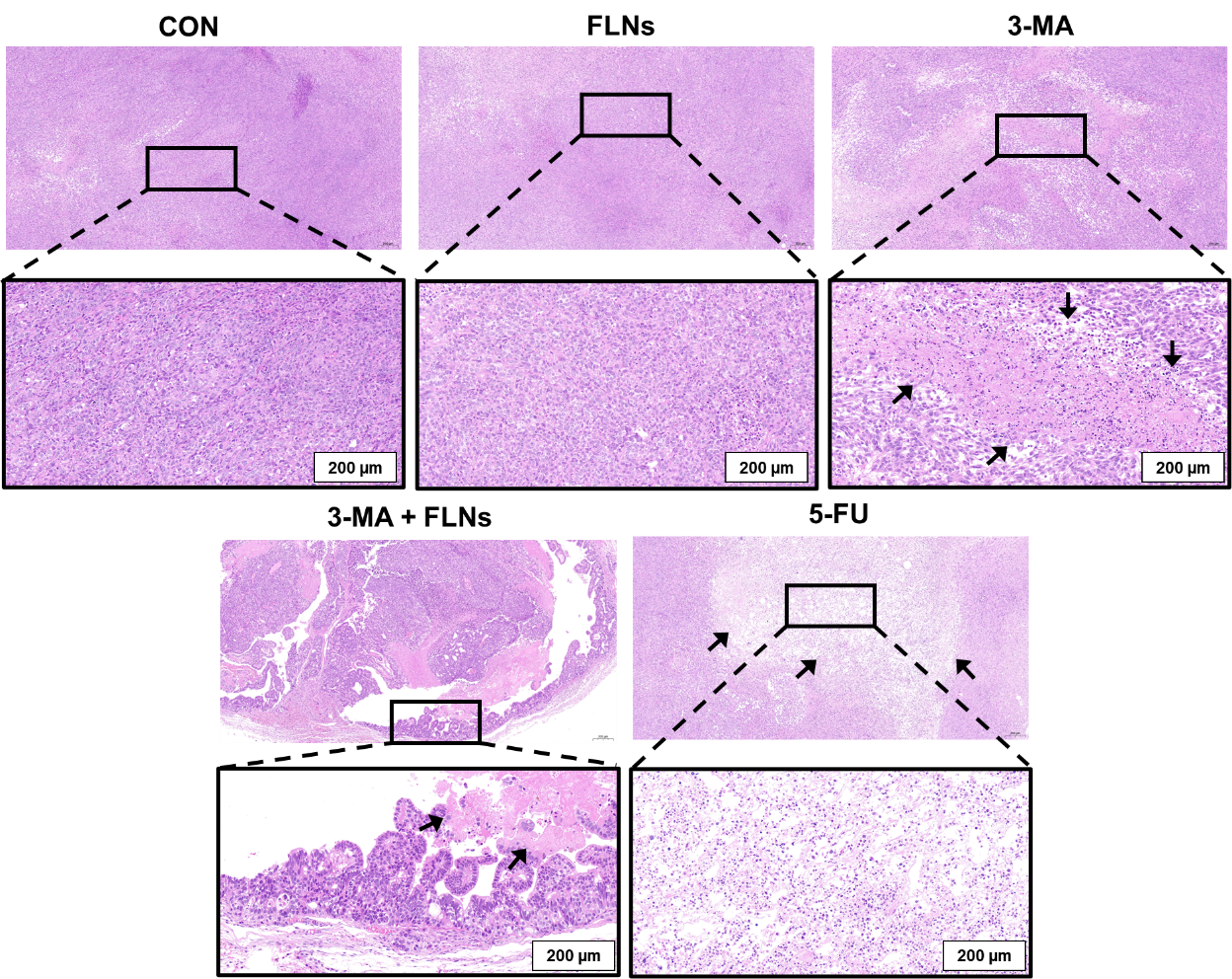
**

**Figure S28.** Representative picture of hematoxylin and eosin (HE) staining of tumor tissue. The black arrows indicate the necrotic area of the tumor, and the black box indicates the local zoom area. Data are presented as mean ± SD; n=3 independent experiments. Abbreviations: CON (Control group), FLNs (Food lipid nanoparticles), 3-MA (3-MA treatment), 3-MA+FLNs (3-MA and FLNs combined treatment), FU (5-fluorouracil).

**
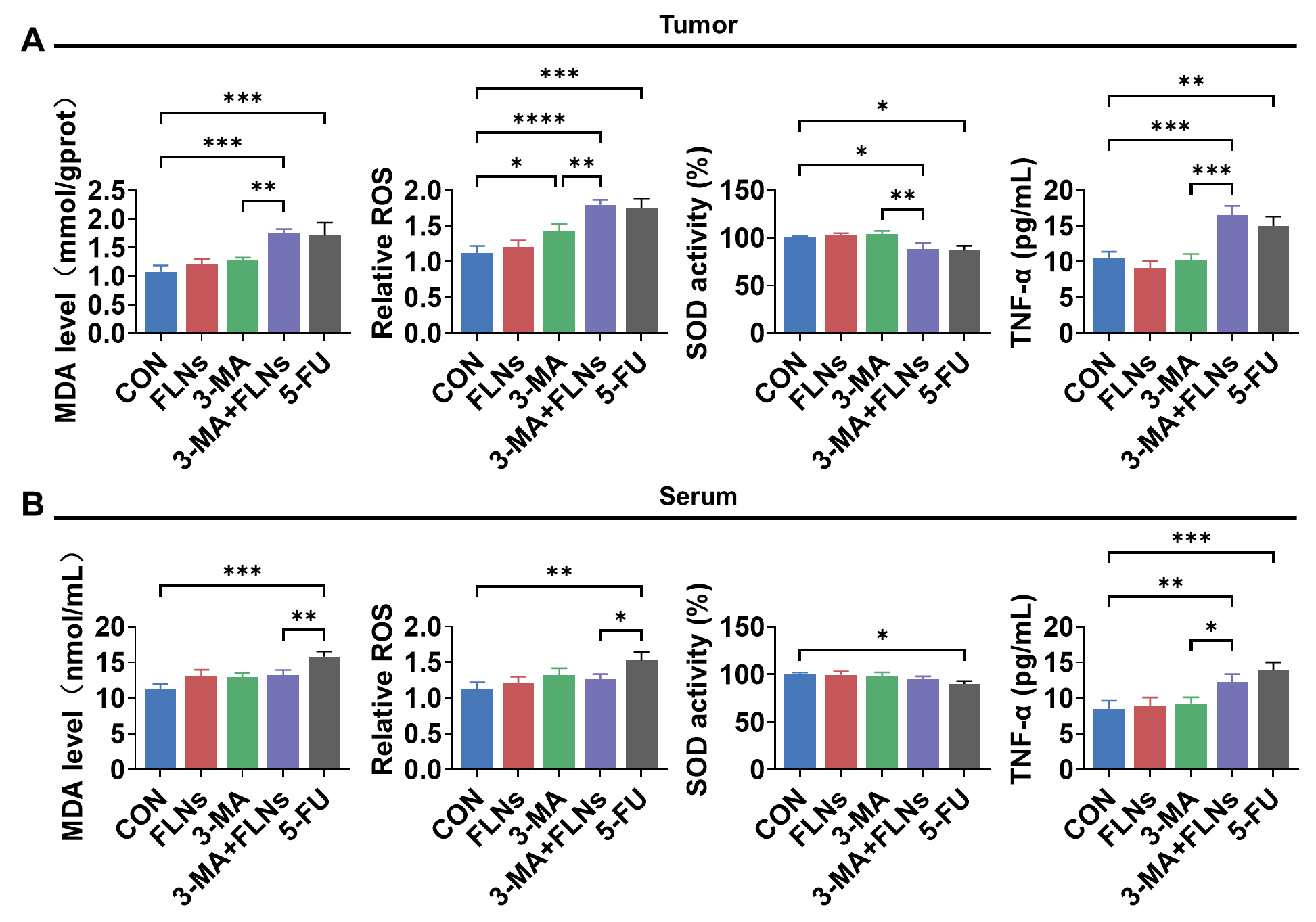
**

**Figure S29.** Effects of FLNs on oxidative stress and inflammatory factors in mice. A, Detection of oxidative stress and inflammatory factors in mice subcutaneous tumors. B, Detection of oxidative stress and inflammatory factors in serum of mice. **P* < 0.05; ***P* < 0.01; ****P* < 0.001 and *****P* < 0.0001. Data are presented as mean ± SD (n=3).


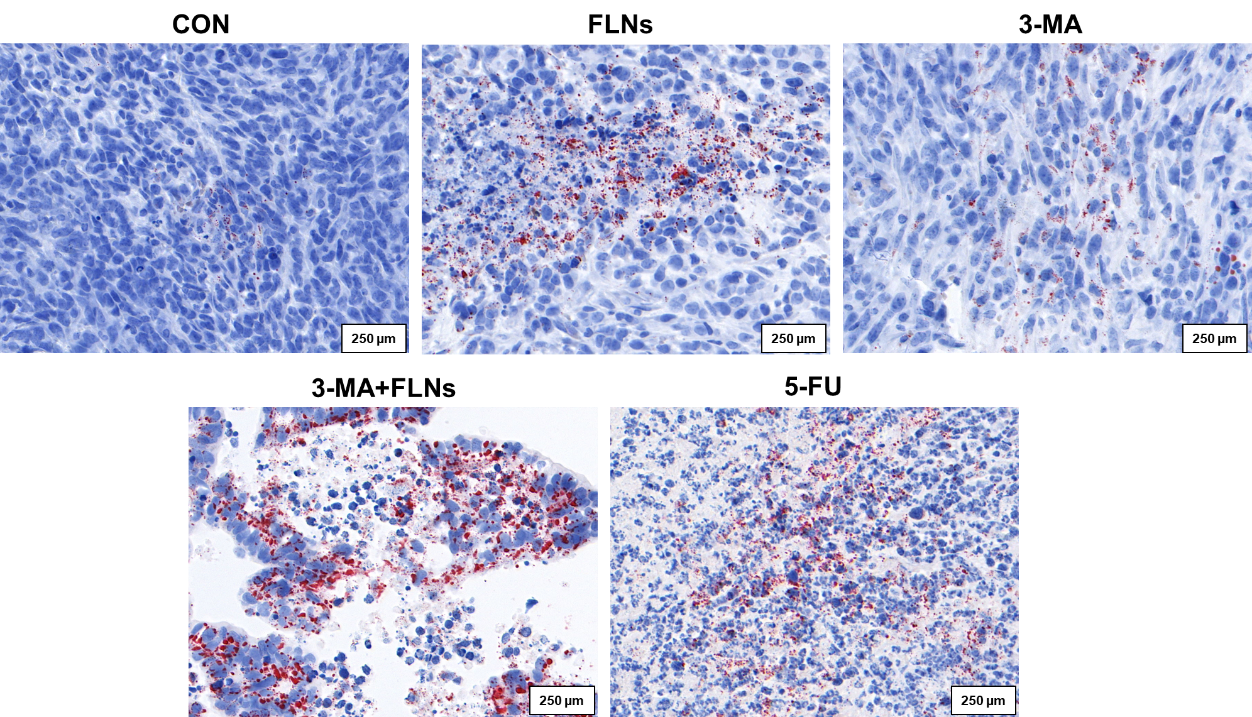


**Figure S30.** The changes of lipid droplets in tumor tissues were observed by oil red O staining. Data are presented as mean ± SD; n=3 independent experiments. Abbreviations: CON (Control group), FLNs (Food lipid nanoparticles), 3-MA (3-MA treatment), 3-MA+FLNs (3-MA and FLNs combined treatment), FU (5-fluorouracil).


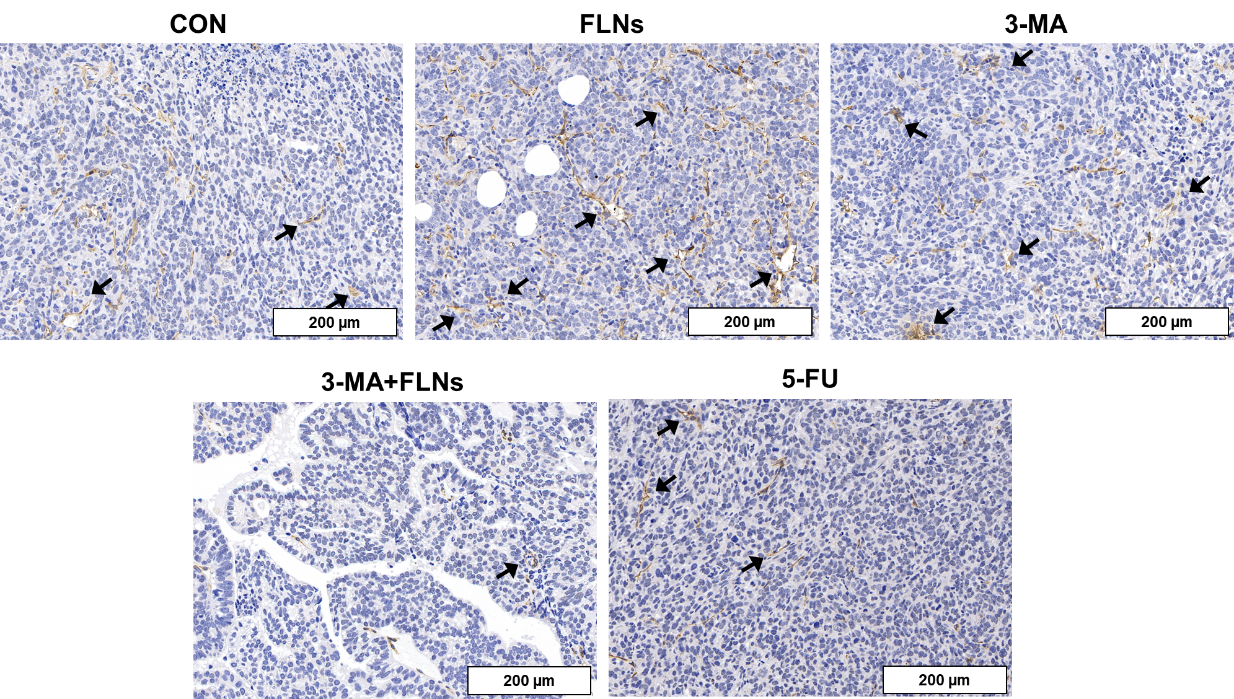


**Figure S31.** Immunohistochemical staining of CD31; the expression of CD31 was determined by immunohistochemistry, and positive expression was identified as yellow or brown granules on the cell membrane, the black arrow indicates positive expression. Data are presented as mean ± SD; n=3 independent experiments. Abbreviations: CON (Control group), FLNs (Food lipid nanoparticles), 3-MA (3-MA treatment), 3-MA+FLNs (3-MA and FLNs combined treatment), FU (5-fluorouracil).


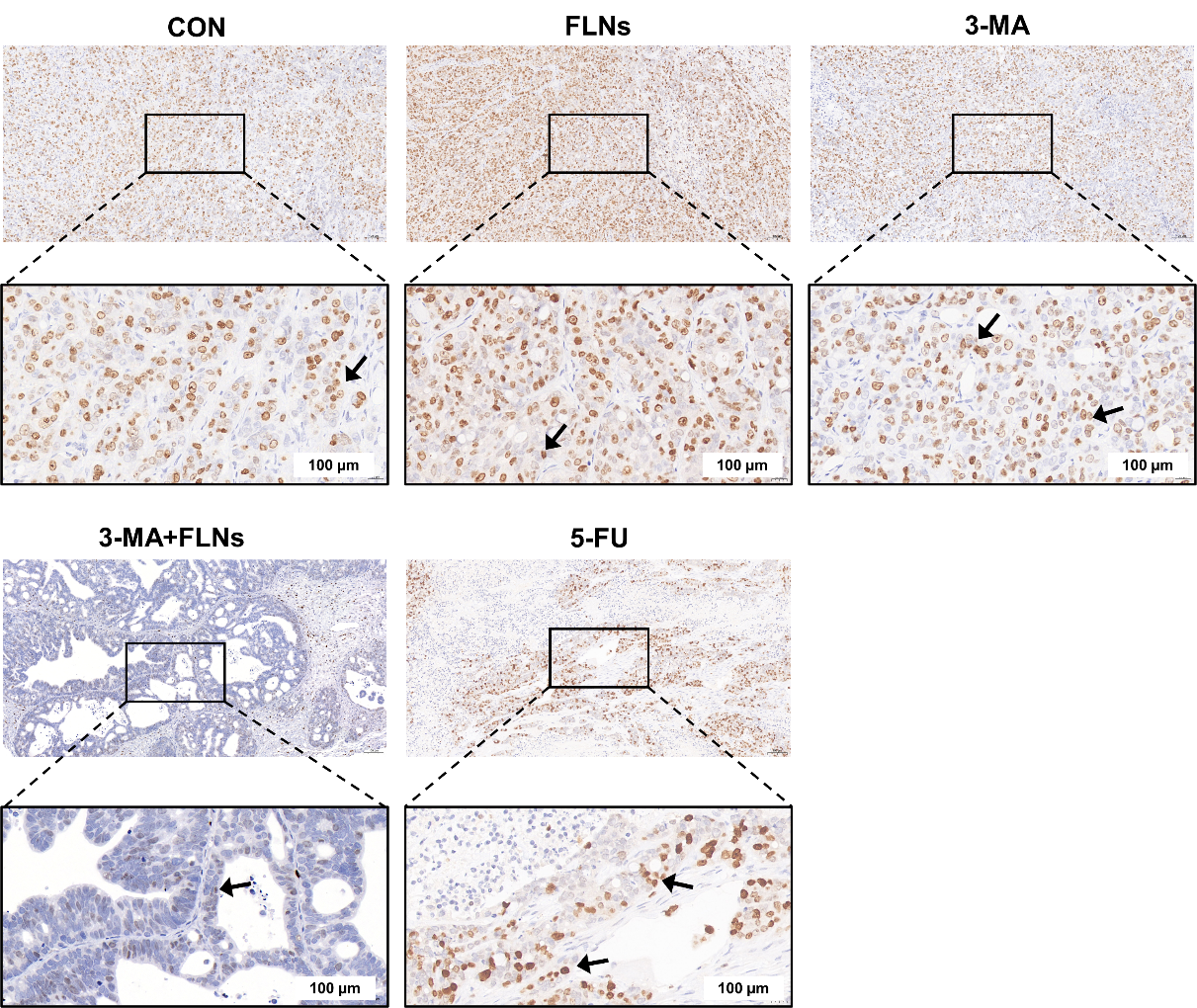


**Figure S32.** Immunohistochemical staining of Ki67; Ki67 is nuclear positive, and the brown dot in image shows Ki67 protein expression, the black arrow indicates positive expression. Data are presented as mean ± SD; n=3 independent experiments. Abbreviations: CON (Control group), FLNs (Food lipid nanoparticles), 3-MA (3-MA treatment), 3-MA+FLNs (3-MA and FLNs combined treatment), FU (5-fluorouracil).

**
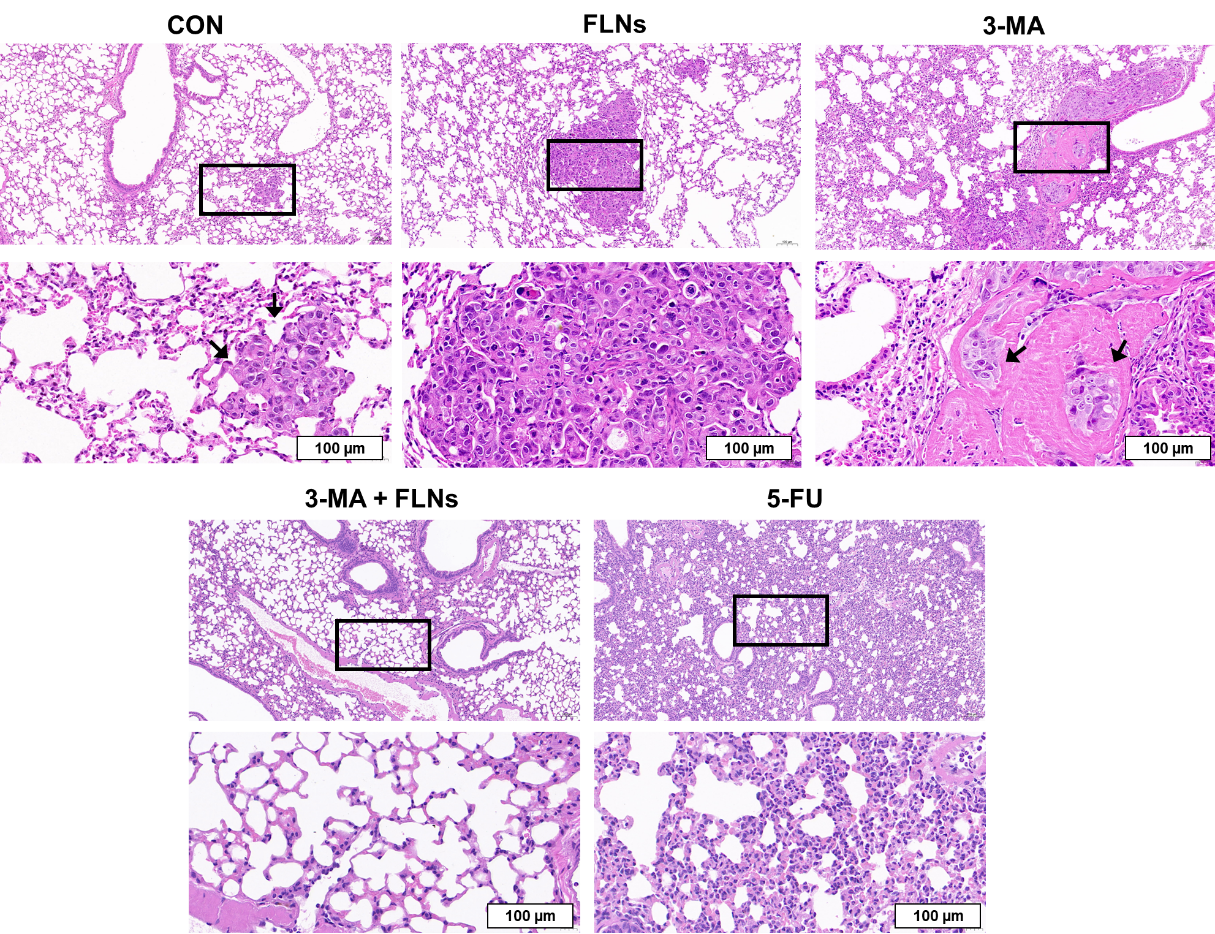
**

**Figure S33.** Representative picture of HE staining of lung tissue. Inside the black rectangle are tumor cells. Data are presented as mean ± SD; n=3 independent experiments. Abbreviations: CON (Control group), FLNs (Food lipid nanoparticles), 3-MA (3-MA treatment), 3-MA+FLNs (3-MA and FLNs combined treatment), FU (5-fluorouracil).

**
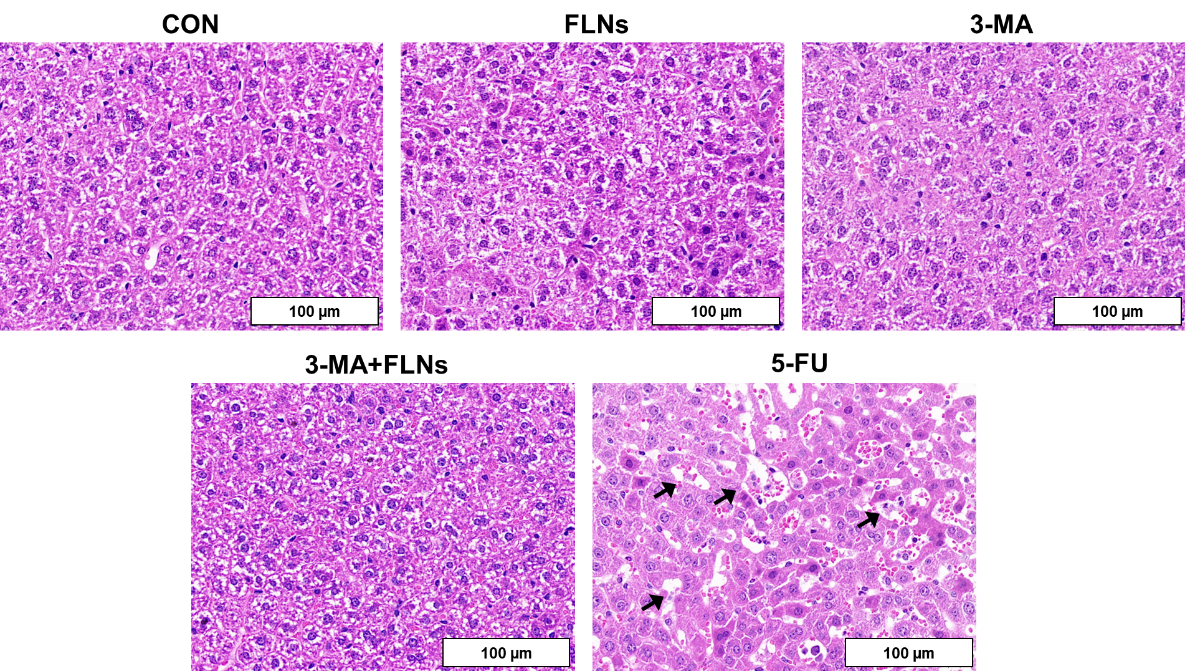
**

**Figure S34.** Representative picture of hematoxylin and eosin (HE) staining of liver tissue. Black arrows indicate areas of edema. Data are presented as mean ± SD; n=3 independent experiments. Abbreviations: CON (Control group), FLNs (Food lipid nanoparticles), 3-MA (3-MA treatment), 3-MA+FLNs (3-MA and FLNs combined treatment), FU (5-fluorouracil).


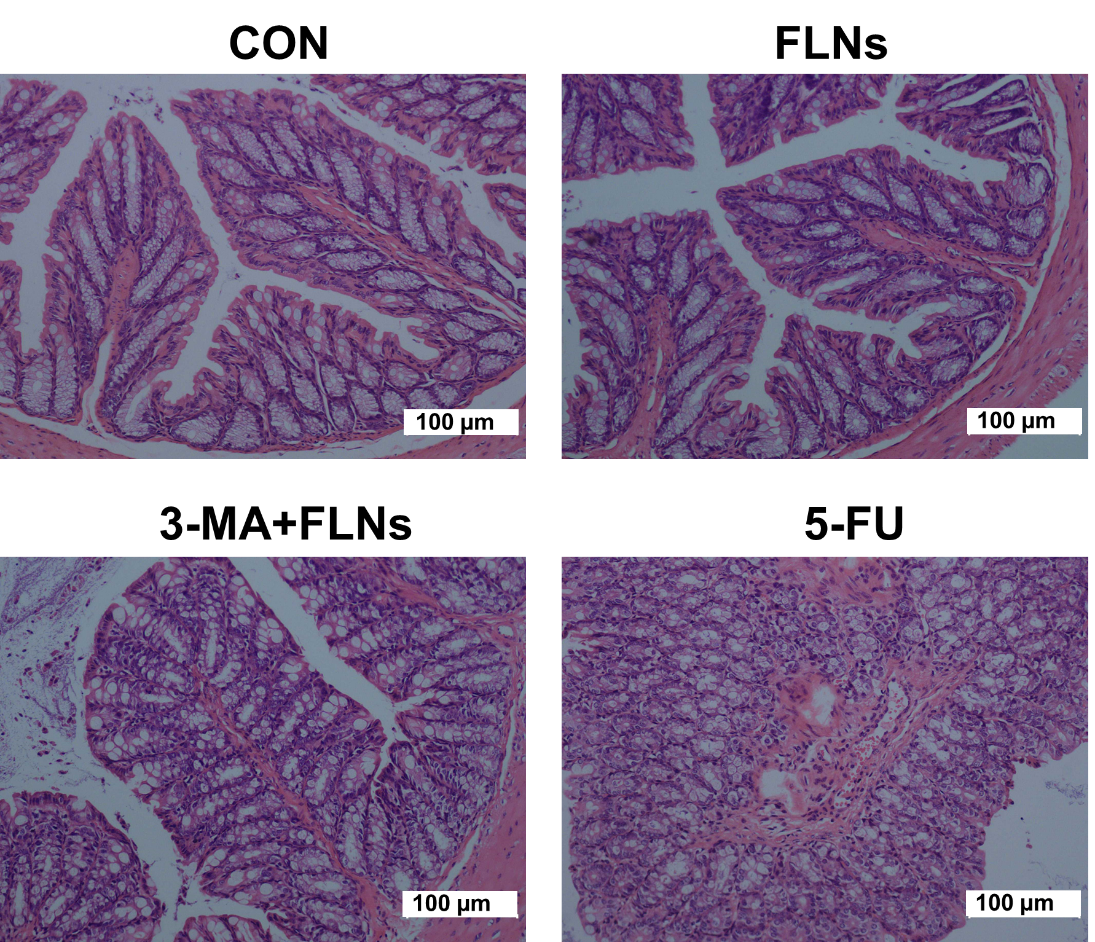


**Figure S35.** Representative picture of hematoxylin and eosin (HE) staining of colon tissue. Data are presented as mean ± SD; n=3 independent experiments. Abbreviations: CON (Control group), FLNs (Food lipid nanoparticles), 3-MA+FLNs (3-MA and FLNs combined treatment), FU (5-fluorouracil).


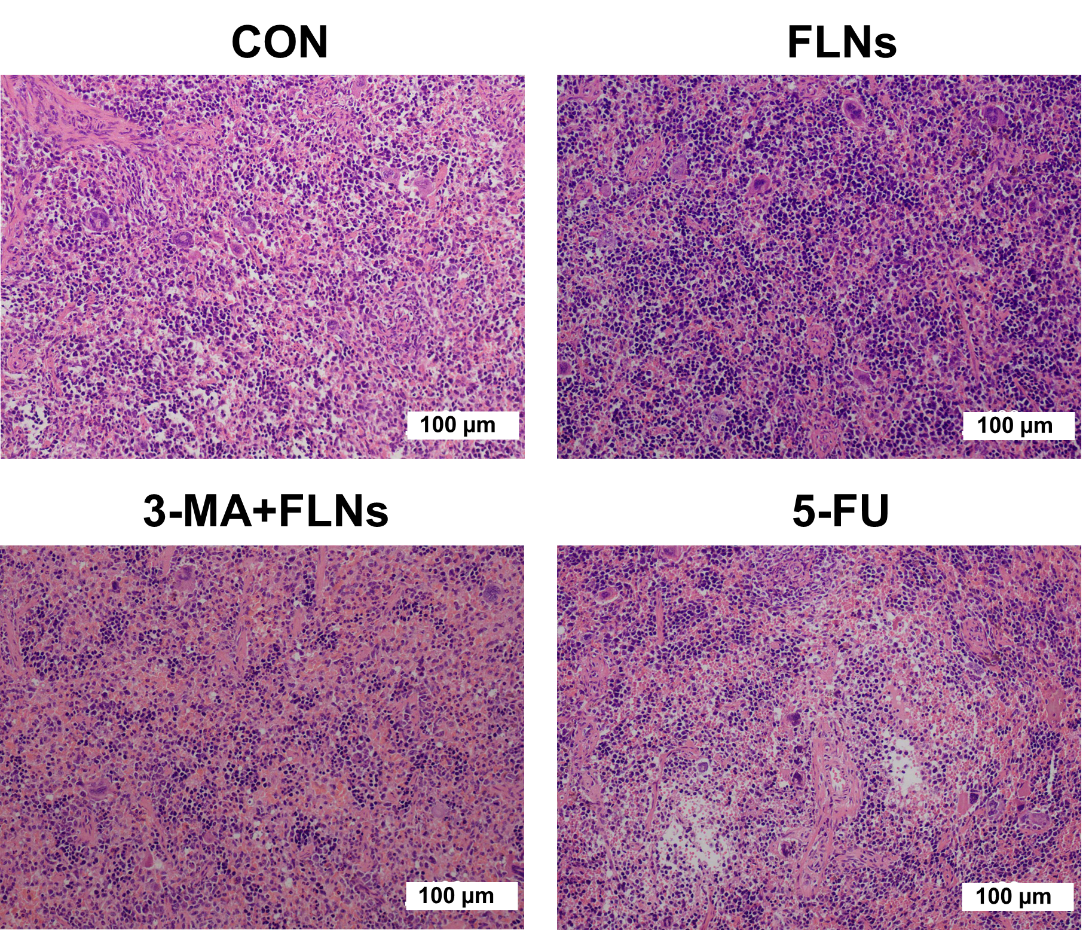


**Figure S36.** Representative picture of hematoxylin and eosin (HE) staining of spleen tissue. Data are presented as mean ± SD; n=3 independent experiments. Abbreviations: CON (Control group), FLNs (Food lipid nanoparticles), 3-MA+FLNs (3-MA and FLNs combined treatment), FU (5-fluorouracil).


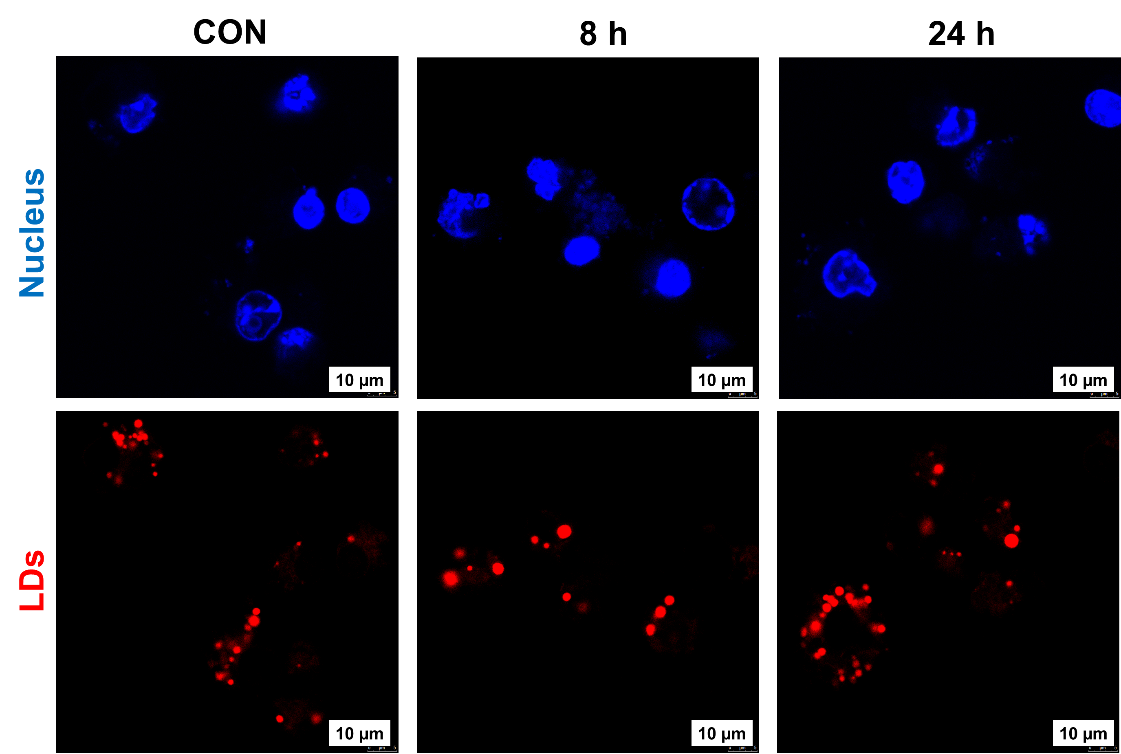


**Figure S37.** Changes of intracellular lipid droplets in RAW264.7 cells treated by FLNs for 8 h and 24 h. Blue fluorescence indicates DAPI-stained nuclei; Red fluorescence indicates Bodipy-stained LDs. Data are presented as mean ± SD; n=3 independent experiments.

**Captions for Movie S1-S3**

Movie S1. CLSM observation of FLNs (red) and LDs (green) co-localization

Movie S2. CLSM observation of FLNs (red) and lysosomes (green) co-localization

Movie S3. CLSM observation of mRFP-GFP-LC3 puncta

**Supplementary materials and methods**

**Preparation and characterization of FLNs**

Oil phase preparation: Mix 1 g of monoglyceride (Aladdin; Shanghai) with 3 g of soybean oil (Mackli; Shanghai) at 80℃ and stir for 20 min to obtain the oil phase. Aqueous phase: Use 200 mL of phosphate-buffered saline (PBS, pH 7.4, 10 mM) as the aqueous phase.

Preparation of FLNs: Slowly add the aqueous phase to the oil phase while maintaining continuous stirring; then subject the mixture to high-speed shearing for further mixing (7000 rpm, 5 min; T25, IKA-Werk, Staufen, Germany); subsequently, the pre-mixed sample is subjected to high-pressure homogenization (AH NANO, ATS, China) under the following conditions: homogenization pressure of 800 bar, temperature of 25℃, and 3 passes. with a homogenization pressure of 400 bar.

The sample was adsorbed onto carbon film on 230 mesh copper grids (Beijing Zhongjingkeyi Technology Co., Ltd) and air-dried for 10 min. FLNs were observed by TEM as a direct measure of their droplet size and shape. The size, PDI, and zeta potential of FLNs were recorded by dynamic light scattering technology (DLS, Malvern Instruments, Malvern, UK).

**Cell lines and culture conditions**

Human colon cancer cells HT-29, Caco-2 were purchased from Procell Life Science & Technology Co. Ltd (Wuhan, China). Hela cells were provided by Prof. Ziyun Wu, who works at Shanghai Jiao Tong University. The cells were routinely cultured in DMEM medium supplemented with 20% fetal bovine serum (FBS) and 1% penicillin-streptomycin-amphotericin. HUVEC (BH0128) was purchased from Nexell (Yuchun Biotechnology Co., Ltd). The cells were cultured in HUVEC complete medium (CCZY001, Nexell). All cells were cultured in a humidified 5% CO_2_/95% atmosphere incubator at 37℃.

AMPK small interfering RNA (siAMPK) and negative control (siNC) were synthesized by Santa Cruz Biotechnology and then transfected into Caco-2 cells according to the manufacturer’s instructions.

**Cell survival assay**

To assess the effect of FLNs on cell viability, cells were grown in 96-well plates (Corning Inc.) at 2 × 10^3^ cells per well with or without 3-MA pre-treatment and exposed to FLNs. After 2 h, 4 h, 8 h, and 24 h, the cells were incubated with 10% Cell Counting Kit-8 (CCK-8; HY-K0301) solution at 37 ℃ for 2 h, and then the absorbance was measured at 450 nm using a microplate reader.

Cell survival rate =[(As-Ab)/(Ac-Ab)] × 100%

As: test well absorbance (including cells, culture medium, CCK-8 solution and drug solution); Ac: control well absorbance (including cells, culture medium, CCK-8 solution, without drugs); Ab: blank well absorbance (including medium, CCK-8 solution, excluding cells and drugs).

**Cellular uptake experiment**

The cellular uptake and intracellular distribution of FLNs in cells were observed and compared by CLSM. Cells (4×10^4^ cells/well) were seeded on round glass cover-slips placed into 12-well plates and incubated for 24 h. Then, the cells were incubated in the fresh culture medium containing FLNs (Nile red labeled). The cells were washed three times with 4℃ PBS, fixed with 4% paraformaldehyde for 30 min, and then stained with DAPI. Finally, the samples were detected by confocal laser scanning microscope (TCS SP2; Leica, Welzlar, Germany) using the Leica Application Suite X, with a 100× oil-immersion objective (HCX PL APO CS ×100 1.4 oil; Leica). The pictures were obtained in a format of 1024 ×1024 pixels. The absorption rate was analyzed using flow cytometry (Beckman Cytoflex, Beckman Coulter Co., Brea, CA, USA). The single-cell population was gated in a plot of FSC versus SSC after excluding cell debris and doublets, and a histogram from the Nile red channel for the single-cell population was obtained and analyzed using FlowJo (version X, FlowJo LLC).

**Endocytosis inhibition test**

Five different blocking reagents (NaN_3_, Chlorpromazine, Nystatin, and Cytochalasin B), with various inhibition mechanisms, were used to analyze the specific mechanism of FLNs, which was involved in the cellular uptake. CLSM and FCM were utilized to investigate the effect of different endocytosis inhibitors on the uptake of FLNs by cells. The inhibitors used are as follows: 20 µg/mL of NaN_3_ to inhibit energy-dependent endocytosis, 20 µg/mL of quercetin (Q; KKL MED) to inhibit clathrin-/caveolin-independent endocytosis; 10 µg/mL of Chlorpromazine (CPZ; Sigma-Aldrich), to inhibit clathrin-dependent endocytosis; 30 µg/mL of Nystatin (NY; Sigma-Aldrich) to inhibit caveolae-dependent endocytosis; 10 µg/mL Cytochalasin B (CB; Sigma-Aldrich) to inhibit phagocytosis-dependent endocytosis.

**Cell proliferation with Alamar Blue Assay**

Cells were seeded into 96-well plates and cultured until they reached an appropriate growth stage, with 70~80% confluence. Subsequently, the cells were treated with FLNs, So, M, and So+M for a duration of 24 h. Following the treatment, the culture medium was removed, and each well was washed three times with phosphate-buffered saline (PBS). Then, 10 μL of Alamar Blue reagent was added to each well, after which the plates were incubated for an additional 2 h. Fluorescence was measured using a fluorescence microplate reader, with an excitation wavelength ranging from 530 to 560 nm and an emission wavelength set at 590 nm. The relative fluorescence units (RFU) were recorded.

**Observation of cell ultrastructure under transmission electron microscope**

Cell samples in vitro were prefixed in 2.5% glutaraldehyde for 24 h at 4℃. After the samples were washed 4 times with 0.1 M phosphate buffer (PB, 10 min each), they were postfixed in 0.2 mL 1% osmic acid for another 1 h at 24±2℃. The cells were then washed 2 times with 0.1 M PB (10 min per wash) and then washed 2 times with ddH_2_O, stained with 1% uranyl acetate for 1 h at 24±2℃, followed by three washes with ddH_2_O (15 min each). Samples were dehydrated in 50%, 70%, 90% ethanol for 10 min, and 90% ethanol solution: 90% acetone (1:1) for 10 min, followed by 100% acetone (3 times, 8 min each). Samples were gradually embedded with 3 mixtures of acetone and EPON812, ratios of 1:1 (1 h), 1:2 (1 h), and 1:3 (overnight) at 24±2℃, and subsequently embedded in pure EPON812 for 48 h at 60℃. The polymerized cell samples were sectioned and stained with 1% uranyl acetate for 20 min, followed by lead citrate for 5 min. 100 nm-thick ultra-thin sections were prepared. Ultrathin sections were observed under a transmission electron microscope (Tecnai G^2^ Spirit BioTWIN) with an acceleration voltage of 120 kV.

**ROS level detection**

ROS levels in cells after FLNs treatment were measured using the 2,7-dichlorofluorescin diacetate (DCFH-DA) probe (S0033S, Beyotime). Then, 1 × 10^4^ cells were cultured in 96-well plates for 24 h and then treated with FLNs, after which cells were washed three times with PBS before incubation in 200 μL serum-free medium with DCFH-DA (1000:1) for 20 min at 37℃. Then, cells were washed three times with serum-free medium, and fluorescence was measured at 485 nm excitation and 520 nm emission using a microplate reader (SpectraMaxi D3). Samples without intervention were used as negative controls. ROS levels of treated groups were expressed as relative fluorescence intensity compared to the control group.

**MDC staining**

Monodansylcadaverine (MDC) staining of autophagic vacuoles was performed for autophagy analysis. Autophagic vacuoles were labeled with 0.05 mmol/L MDC in PBS at 37 ℃ for 10 min. After incubation, the cells were washed three times with PBS and immediately analyzed under a CLSM (TCS SP8 STED 3X, excitation: 335 nm, emission: 512 nm) with a 100 × oil immersion objective. Fluorescence of MDC was excited by a wavelength of 488 nm and detected at 493 to 558 nm. The results are presented as the percentage of cells with puncta and/or blue spots.

**Measurement of autophagic flux using the mRFP-GFP-LC3 lentiviral vector**

The GFP and mRFP expressed in mRFP-GFP-LC3 tandem fluorescent protein lentvirus (Hanbio) are used to label and track LC3, and the weakening of GFP can indicate the fusion of lysosome and autophagosome to form autophagolysosome (due to the sensitivity of GFP fluorescent protein to acidity, the GFP fluorescence is suppressed after the fusion of autophagosome and lysosome, and only red fluorescence can be detected at this time). The cells were infected (amount of virus added per well (μL) =MOI* Number of cells/virus titer (PFU /mL) ×1000). It was filled to culture volume 4 h after lentivirus infection. On the second day after infection (about 24 h), the culture medium containing the virus was removed, replaced with fresh complete culture medium, and continued to culture at 37℃. 48 h after infection, the expression efficiency of GFP was observed by fluorescence microscopy, and the cell lines with stable transduction were screened by fresh and complete culture medium containing an appropriate concentration of Puromycin. The final concentration range of Puromycin was 1-10 μg/mL. At least 5 clones of infected and screened cells were selected for cell expansion, and Puromycin was continued for screening. Stable cell lines with moderate expression were selected, screened, and transmitted continuously for 3 generations, and frozen and preserved.

**Enzyme-linked immunosorbent assay (ELISA)**

IL-8 and Caspase-3 were determined using an ELISA kit (Beyotime) according to the manufacturer’s instructions. The cells were lysed in lysis buffer, and the supernatants were collected with centrifugation (3000 g for 5 min at 4℃), then the supernatants were co-incubated with working solution in 96-well plates for 30 min at 37℃. A microplate reader was used to monitor color development, and the optical density was read at 450 nm.

**Western blotting**

FLNs-treated cells were collected and lysed with RIPA lysis buffer on ice for 30 min. After centrifugation (10,000 g, 10 min) at 4℃, the proteins were obtained and the corresponding concentrations were quantified using BCA (Bicinchoninic acid) protein assay kit (P0012S, Beyotime). Subsequently, approximately 40 μg of denatured protein samples, which were mixed with 4 × loading buffer and boiled for 5 min, were electrophoresed by 12% SDS-PAGE and transferred into polyvinylidene difluoride membranes under a Semi-Dry Transfer Cell (Bio-Rad, USA). The membranes were sealed with BSA (5%, w/v) for 3 h and cultured with the primary antibodies for 16 h at 4℃. The membranes were washed with Tris-buffered saline Tween three times to remove the superfluous primary antibodies, after which they were probed with the corresponding HRP-conjugated secondary antibodies for 1.5 h. The protein expressions were visualized using an enhanced chemiluminescence (ECL) kit (Bio-Rad, USA). The bands were captured using Quantity One software and their relative amounts were evaluated after normalization with the corresponding β-actin values. The primary antibodies used in this study include the following (antibody sources, catalog numbers, and working dilutions are indicated in parentheses).

**Table 1** Primary antibodies used in this study

| Primary antibodies | Sources | Catalog numbers | Dilutions |
| --- | --- | --- | --- |
| p-AMPK | Rabbit | AA393 | 1:1000 |
| AMPK | Rabbit | ab3759 | 1:1000 |
| p-mTOR (Ser 2448) | Rabbit | sc-101738 | 1:1000 |
| mTOR | Rabbit | ab131538 | 1:1000 |
| Anti-ULK1 (phospho S555) | Rabbit | ab229537 | 1:1000 |
| Anti-ULK1 | Rabbit | (ab240916) | 1:1000 |
| Phospho-PI3 Kinase Class III | Rabbit | #13857 | 1:1000 |
| Beclin 1 | Rabbit | ab207612 | 1:2000 |
| LC3 | Rabbit | ab192890 | 1:1000 |
| Bax | Rabbit | ab32503 | 1:3000 |
| Cyt-c | Rabbit | ab18738 | 1:1000 |
| Bcl-2 | Rabbit | ab32124 | 1:1000 |
| HSP60 | Rabbit | ab46798 | 1:5000 |
| β-actin | Rabbit | BM3873 | 1:5000 |
| Goat anti-Rabbit IgG HRP | Goat | GTX213110-01 | 1:3000 |

**Mitochondrial extraction**

Mitochondria were extracted by adopting the mitochondrial extraction kit (Beyotime® Biotechnology, Hangzhou, China). Cells were collected from the cell culture plate by trypsin digestion. After centrifugation at 1400 g for 5 min, the cells were collected and washed twice with PBS and then incubated with mitochondrial extraction reagent for 15 min. The nuclei and broken cell debris were sedimented through centrifugation at 1000 g for 10 min at 4℃. The suspension was transferred to another tube and centrifuged at 12,000 g for 8 min at 4℃. Supernatant (cytosol-enriched fraction) and sediment (mitochondria) were collected separately. Mitochondrial and cytoplasmic Cyt-c protein expression was detected by Western blotting, respectively.

**Immunofluorescence**

After treatment, the supernatant was discarded and cells were washed by PBS. Cells were fixed with 4% paraformaldehyde (Sigma-Aldrich), reacted with primary antibodies, washed with PBS, reacted with corresponding FITC-conjugated secondary antibodies (F-2765, Thermo Fisher Scientific), and counterstained with DAPI. Immunofluorescence was observed with a CLSM (TCS SP8 STED 3X). Confocal images were further processed with Leica Application Suite X software and ImageJ (National Institutes of Health, USA).

**Measurement of mitochondrial membrane potential**

The effects of FLNs on the ∆Ψm of cells were further verified by a JC-1 (5, 5′, 6, 6′‐tetrachloro‐1, 1′, 3, 3′‐tetraethylbenzimidazolylcarbocyanine iodide, Thermo Fisher Scientific) mitochondrial membrane potential assay kit. FLNs-treated cells were collected and stained with JC-1 solution based on the manufacturer's instructions. Healthy mitochondria, which have a high potential, showed a high intensity of red fluorescence at 594 nm and a low intensity of green fluorescence at 488 nm; damaged mitochondria showed a low intensity of red fluorescence at 594 nm and a high intensity of green fluorescence at 488 nm. The ratio of red/green intensity was measured to evaluate the function of isolated mitochondria from each group.

**TUNEL assay**

Detection of DNA fragments in situ using terminal deoxyribonucleotidyl transferase-mediated dUTP-digoxigenin nick end labeling (TUNEL) assay kits (CC1731, G-CLONE) was applied to investigate active cell death. Cells were fixed with 4% paraformaldehyde, rinsed with PBS. Intracellular DNA fragments were then labeled by exposing the cells to TUNEL reaction mixture for 1 h at 37℃ in a humidified atmosphere and counterstained with 4,6-diamidino-2-phenylindole (DAPI) with protection from light. Immunofluorescence is observed with a CLSM (TCS SP8 STED 3X). Five fields in each section were randomly selected for counting the number of TUNEL-positive cells with green fluorescence and the number of total cells with blue fluorescence. The rate of apoptosis was expressed as the ratio of apoptotic cells to normal cells.

**Apoptosis assay**

Apoptosis detection was performed with the Annexin V-FITC/PI apoptosis Detection Kit (Beyotime). FLNs were exposed for 2, 4, 8, and 24 h after pretreatment with or without 3-MA (KKL MED), cells obtained by trypsin (Gibco) digestion and centrifugation (1400 g×5 min). Cells were collected and washed with PBS 3 times and then resuspended in 200 µL of binding buffer. 5 µL of Annexin V-FITC stock solution was added to the cells and incubated for 10 min at room temperature, avoiding exposure to light. Then, 10 µL propidium iodide (PI) was added. Approximately 1×10^4^ cells were analyzed in each of the samples. The apoptosis of cells was measured using a flow cytometer (NovoCyte, Agilent). Data were analysed with the FlowJo software.

**Scratch-wound treatments**

To induce migration, a scratch or multiple scratches using pipette tips were introduced into a six-well cell culture plate. The cells were further cultured under serum starvation for 30 min and were subsequently cultured for an additional indicated time.

The change in wound width before and after treatment was observed with a light microscope. The wound width was measured by Image J software, and the wound healing rate was calculated with the following equation: $Wound healing rate=(W0-W)/W0$ . W0 and W are the wound width of the sample at 0 h and 48 h, respectively.

**Transwell migration assays and automated scratch migration assays**

For migration studies, a standard assay was used to determine the number of cells that traversed a porous polycarbonate membrane in response to FLNs using the Cytoselect 24-well cell migration assay (Cell Biolabs). 1.5 × 10^5^ and 2.5 × 10^5^ cells per well were seeded in an upper chamber in serum-free media for the migration and invasion assay, respectively. The lower chamber was filled with media containing 10% FBS with/without FLNs. After 24 h, cells passing through the polycarbonate membrane were stained and counted according to the manufacturer’s instructions.

**Exosome isolation and procedures**

Differential centrifugation (Beckman ultracentrifuge with an SW28 fixed-angle rotor) was performed to isolate exosomes from the conditioned medium. Initial spins consisted of a 1,000 g spin for 10 min, a 2,000 g spin for 10 min, and a 10,000 g spin for 30 min. The supernatant was retained each time. The supernatant was then spun at 100,000 g for 70 min, and the pellet was resuspended in PBS to dilute remaining soluble factors, followed by another centrifugation at 100,000 g for 70 min. The final pellet contained the exosomes, which were resuspended in tissue culture media. This protocol is based on previous exosome isolation methods ^15^.

**HUVEC tube formation on Matrigel**

The tube formation assay is a rapid and quantitative method for determining genes or pathways involved in angiogenesis. The principles underlying this assay are that endothelial cells retain the ability to divide and migrate rapidly in response to an angiogenic signal ^44,45^. Further, endothelial cells are induced to differentiate and form tube-like structures when cultured on a matrix of basement membrane extract. These tubes contain a lumen surrounded by endothelial cells linked together through junctional complexes. Tube formation occurs quickly, with most tubes forming in this assay within 2-6 h, depending on the quantity and type of angiogenic stimuli. Matrigel was used to test the effects of extracellular vesicles on in vitro vascular tube formation as described. Different groups of extracellular vesicles were added to HUVEC plated on Matrigel in endothelial basal medium. Images were captured after 4 h of incubation using a brightfield microscope and analyzed using the Image J software plugin-Angiogenesis analyzer.

**In vivo subcutaneous tumor model**

All in vivo experimental protocols were approved by the Animal Protection Committee of Shanghai Jiao Tong University (Ethical Number: A2023175-001). Viable Caco-2 cells (5×10^6^/200 µL) were subcutaneously injected into the left axilla of 8-week-old male M-NSG mice (6 mice per group). Tumor volume was measured every 3 days for 3 weeks and calculated by the following formula: (short diameter)^2^ × (long diameter)/2. Tumor-bearing mice with a tumor volume of around 100 mm^3^ were received with different treatments by intratumoral injection. Every other day, intratumoral injection of PBS (100 μL), FLNs (100 μL; 1 mg/mL), 3-MA (100 μL; 2 mg/mL), FLNs (100 μL; 1 mg/mL) +3-MA (100 μL; 2 mg/mL) was conducted.

**Immunohistochemistry**

IHC for CD31, CD34 and Ki67 was performed on formalin-fixed paraffin-embedded (FFPE) sections using standard protocols. The tissue sections were treated with sodium citrate buffer (pH 6.0) in a microwave oven for 10 min, followed by bench-cooling for 20 min, and the same cycle was repeated for the antigen retrieval. Endogenous peroxidase activities of the tissue were blocked by incubating the tissue sections with 3% H_2_O_2_ in methanol for 30 mins. The nonspecific tissue reactions were prevented by incubating the tissue sections with 10% serum for 10 min. Tissue sections were then incubated with the primary antibody (Ki67). Tissue sections with bound primary antibody were then incubated with the appropriate secondary antibody. The positive staining of CD31, CD34 and Ki67 was brown-yellow. CD31 and CD34 were tumor microvascular markers, and Ki67 was expressed in the nucleus.
